# The Effectiveness of Nurse‐Led Transition Care on Post‐Discharge Outcomes of Adult Stroke Survivors: A Systematic Review and Meta‐Analysis

**DOI:** 10.1002/nop2.70140

**Published:** 2025-02-28

**Authors:** Nyagwaswa Athanas Michael, Lilian Teddy Mselle, Edith Mroso Tarimo, Yingjuan Cao

**Affiliations:** ^1^ School of Nursing and Rehabilitation Shandong University Jinan People's Republic of China; ^2^ School of Nursing Muhimbili University of Health and Allied Sciences Dar es Salaam Tanzania; ^3^ Department of Nursing Qilu Hospital of Shandong University Jinan People's Republic of China

## Abstract

**Aim:**

To evaluate the effectiveness of nurse‐led Transition Care (TC) on quality of life, mortality and readmission among adult stroke survivors.

**Design:**

Systematic review and meta‐analysis using the Joanna Briggs Institute (JBI) methodology for systematic reviews of effectiveness.

**Methods:**

Databases were searched from 1st January 2000 to 31st December 2023. Search was done in Medline, Science Direct, Scopus, EBSCOhost, Web of Science and Cochrane Library and Central Registry of Clinical Trials. The randomised controlled trials that assessed the effectiveness of nurse‐led TC for adult stroke survivors were included. The primary outcome was 1‐year quality of life, whereas the secondary outcomes were mortality and readmission. Two independent reviewers performed data extraction and quality appraisal by using the JBI methodology. Random effect models and pre‐specified subgroup analyses were performed using Review Manager Software Version 5.3. and STATA Version 17.

**Results:**

Of 1093 studies retrieved from database search, only 18 studies met the review inclusion criteria. The pooled results show that stroke survivors who were allocated to nurse‐led TC programs had a 1‐year better quality of life compared to those allocated to usual care (12 studies; SMD = 0.52; 95% CI = 0.20, 0.85; *p = 0.002*; *I* ^2^ = 94%). However, no significant differences in the odds of mortality and readmission were observed.

No patient or public contribution.

## 1 Introduction

Globally, stroke is the third‐leading cause of death and disability, with significant impacts in World Bank low‐ and middle‐income countries (Husseini et al. 2023). The current prevalence, incidence, mortality, and disability‐adjusted life‐years (DALYs) from stroke are estimated to be 101, 12.2, 6.6 and 143 million, respectively (Walker 2022; Tawa et al. 2021). This represents a substantial rise in stroke burden with an 85% rise in prevalence rate, 70% rise in incidence rate, 43% rise in death rate and 32% rise in DALYs in the past two decades (Tawa et al. 2021). Stroke survivors experience physical and cognitive impairment that significantly affect their functional status and quality of life (Husseini et al. 2023).

Addressing the stroke burden requires a multifaceted approach, including preventive strategies targeting risk reduction, rocketing access to acute stroke care, establishing stroke units and improving post‐discharge care (Walker 2022). So far, in several countries, efforts are underway to raise awareness about stroke risk factors, healthy lifestyles, and access to acute stroke care services (Tawa et al. 2021). However, the efforts to address post‐acute care including transition care have not been widely realised in the current initiatives.

The transition from hospital to home represents a critical juncture in the continuum of stroke care. Studies have shown that 1 in 3 stroke survivors experience depression, anxiety, and adjustment disorders during their first year of stroke (Chun et al. 2022). Similarly, about 38% of stroke survivors have low health literacy that impede their ability to follow discharge instructions and advocate for their own care needs. Low health literacy coupled with post‐stroke mood disorders and cognitive impairments increase the risk of medication errors, missed appointments, and recurrent strokes (Flink et al. 2023). Furthermore, financial constraints pose substantial barrier for stroke survivors to access post‐discharge care and support services (Garnett et al. 2022).

To improve stroke survivors' post‐discharge outcomes, a well‐coordinated TC pathway is required. Unfortunately, there is no consensus on the ideal TC pathway or who should coordinate its delivery. In some settings, stroke TC programs are coordinated by nurses, pharmacists, social workers, physicians and a combination of healthcare professionals (Puhr and Thompson 2015). Thus, there is a need for the coordination to be done by a team or group of healthcare professionals who are always on the frontline. Nurses, with their specialised knowledge and patient‐centered approach are uniquely positioned to deliver transition care interventions to address the holistic needs of stroke survivors.

Following the advance of transition care theory by Meleis et al. (2000), nurse‐led TC programs have emerged as promising approaches to address the complex needs of stroke survivors during hospital‐to‐home transitions. Nurse‐led TC programs aim to bridge the gap between hospital‐based care and home‐based care by providing care coordination, early supported discharge and follow‐up services to enhance continuity of care (Joo and Liu, 2021). Studies have shown that nurse‐led TC programs have the potential to enhance post‐discharge quality of life by empowering stroke survivors to actively engage in their self‐care (Leithaus et al. 2022; Burke et al. 2013).

Nurse‐led TC programs involve coordinated and comprehensive support provided by nurses during hospital‐to‐home transition. These programs delivered by skilled nurses facilitate seamless transfer of care, optimise patient outcomes and prevent adverse events. Compared to usual care, nurse‐led TC programs have benefits that go beyond the immediate clinical outcomes of stroke survivors (Berthelsen 2024). Nurse‐led TC programs help patients adhere to treatment plans and follow‐up visits, thereby promoting their independence and self‐management skills. Moreover, effective nurse‐led TC programs reduce adverse events and improve quality of life, resulting in potential cost savings for individuals and healthcare systems (Burke et al. 2013; Kripalani et al. 2019).

However, like other multidisciplinary TC programs (Schwarzbach et al. 2023), the effectiveness of nurse‐led TC programs remains less elucidated. Although some existing original studies have reported positive outcomes associated with nurse‐led TC interventions, others have found no significant differences in patient outcomes. Moreover, there is heterogeneity in model components, intensity, follow‐up duration and outcome measures across studies (O'Callaghan et al. 2022). Such inconsistencies make it difficult to draw definitive conclusions about the effectiveness of nurse‐led TC programs on the post‐discharge outcomes of stroke survivors.

Our initial search in the Cochrane database did not retrieve any systematic review that evaluated the effectiveness of nurse‐led TC programs on the post‐discharge clinical outcomes of stroke survivors. The available systematic reviews evaluated the TC programs led by multidisciplinary teams (Puhr and Thompson 2015; O'Callaghan et al. 2022). Therefore, the current systematic review and meta‐analysis aimed at assessing the effects of nurse‐led TC programs on mortality, readmissions and quality of life among adult patients with stroke within 1‐year after discharge. This evaluation is imperative in generating evidence on nursing care interventions during hospital‐to‐home transition.

## 2 Methods

### 2.1 Protocol Registration

We adopted the Joanna Briggs Institute (JBI) approach for systematic review of effectiveness (Tufanaru et al. 2020). The study protocol has been registered with the international prospective register of systematic reviews (PROSPERO); CRD42024476815. We followed the preferred reporting items for systematic review and meta‐analyses (PRISMA) as a reporting guideline (Page et al. 2021).

### 2.2 Search Strategy

Data sources were searched from 1st January 2000 to 31st December 2023. We searched six databases, namely Medline (Ovid), Science Direct (Elsevier), Scopus (Elsevier), EBSCOhost, Web of Science and Cochrane Library & Central Registry of Clinical Trials (Wiley), to identify English‐language published studies. For unpublished journal articles, we searched in EBSCOhost Open Dissertations through the Shandong University library.

We adopted a comprehensive three‐step approach to electronic literature search. Firstly, one reviewer conducted an initial search on the Medline database by using the keywords; (exp stroke/OR stroke.mp) AND (exp transitional care/OR exp continuity of patient care/OR exp patient discharge/) AND (exp quality of life/OR readmission/OR mortality/). Then, two reviewers jointly checked the abstracts and titles of the retrieved relevant articles for similar keywords and text words. The two reviewers discussed and combined the identified search terms to form the comprehensive search strategy that was modified according to the specific database (Table S1). One reviewer comprehensively searched electronic data sources to find the relevant published studies and grey literature available by 29th March 2024. The search was limited to studies written in English and published between 1st January 2000 and 31st December 2023. Finally, two reviewers independently screened the references of the relevant studies and invited a third reviewer to resolve any disagreement. One reviewer contacted the original authors for additional studies.

### 2.3 Inclusion Criteria

The studies were included based on population, intervention, comparison, outcome and study design (PICOS) criteria. Population: adult stroke survivors transitioning from hospital to home‐based care or rehabilitation, aged 18 years and above who sustained ischemic or hemorrhagic stroke. Intervention: along with usual discharge care, nurse‐led TC program in the intervention group. The TC program was included if it contains a multicomponent interventions such as early supported discharge, education, counselling, rehabilitation skills training and social support system engagement (Burke et al. 2013). The model/program interventions were administered during hospitalisation and continued after discharge. Comparison: usual discharge care and follow‐up. Outcomes: primary outcome was quality of life, whereas secondary outcomes were mortality and hospital readmissions within 1‐year post‐hospitalisation. Study design: only two‐arm parallel randomised controlled trials (RCTs).

Studies were excluded if: (1) stroke survivors had underlying psychiatric, neurodegenerative or organic disorders; (2) TC interventions delivered solely at post‐discharge; (3) the comparison group received usual care per hospital protocol plus more than two intervention components of the intervention group (Burke et al. 2013); (4) outcomes of interest were missing or measured by invalid tools and (5) RCTs with more than two groups.

### 2.4 Data Extraction

The search results from all databases were directly exported to Endnote Version X9. The first reviewer removed all duplicates and then screened the abstracts and titles of retrieved studies. The second reviewer cross‐checked the process and discussed with the first reviewer until consensus was obtained. Then, after repeatedly reading, the two reviewers trained in evidence‐based nursing and epidemiology independently checked the full texts of potential studies versus inclusion criteria. A third reviewer was invited to facilitate consensus when there was disagreement between the two reviewers. All studies that did not meet the reviews' inclusion criteria were excluded with reasons.

The two reviewers independently performed data extraction using the tailored data extraction format from the Joanna Briggs Institute System for the Unified Management, Assessment, and Review of Information (JBI‐SUMARI) (Tufanaru et al. 2020). The two reviewers independently extracted specific study characteristics such as authors' names, article title, year of publication, funder, conflict of interest, journal, study design, methods (data collection method, period and duration), the phenomenon of interest with its confirmation, context/setting and country, participants' characteristics (nature of participants, physiological indicators, sample size and sampling method, age, gender, risk factors, complications and inclusion/exclusion criteria), data analysis plan and conduct and authors' conclusion. Also, the reviewers extracted information related to methodological quality such as randomization, concealment, group baseline characteristics, blinding, intervention fidelity, follow‐up and attrition rates, principle of outcome analysis and outcome measurement (Table S1).

The specific study findings from the primary studies (Table S2) were readmission rates, mortality rates and quality of life of stroke survivors within 1‐year after hospital discharge. The outcome data were extracted with their accompanying numerical values after repetitive reading of the result sections of the included studies. Two reviewers independently searched for and extracted the findings in form of numerical data from the included studies. The extracted findings were exported to excel spreadsheet for organisation. A third reviewer was invited to cross‐check the quality and completeness of data entry.

### 2.5 Quality Appraisal

To ascertain for quality of evidence obtained from this study, the two independent reviewers appraised the selected studies by using the Joanna Briggs Institute's quality appraisal tools for RCTs (13 items) (Tufanaru et al. 2020). These critical appraisal checklists contain questions that assess the methodological congruency and the reporting quality of primary studies. Each question was scored as yes (Y), no (N), unclear (U) or not applicable (N/A). The two independent reviewers resolved their disagreements through an invitation of the third reviewer.

### 2.6 Synthesis Methods

Narrative synthesis by use of texts and tables was used to compare studies that don't offer meta‐analysis. Meta‐analysis was conducted using Review Manager Software Version 5.3 and Stata version 17. For quality‐of‐life outcome, the magnitude of the overall effect size was calculated based on the pooled standard mean difference (SMD). The SMD (Cohen's *d*) of 0.20, 0.50 and 0.80 were considered as small, medium and large effect size. For mortality and readmission outcomes, the overall effect sizes were estimated by using pooled odd ratios (OR).

Due to high heterogeneity, random effect model with inverse variance method was used in all analyses. The Cochran's *Q*‐test (*p* = 0.10) was used to evaluate statistical heterogeneity, whereas the *I*‐squared statistic (*I* ^2^) was used to evaluate the between‐studies heterogeneity. The *I* ^2^ was interpreted as low (25%), moderate (50%), and high heterogeneity (75%) (Tufanaru et al. 2020). In all tests, a *p*‐value of < 0.05 was considered to be statistically significant indicator.

Subgroup analysis was conducted to investigate the effectiveness of nurse‐led TC programs at less than 6 months and 6 months and above. The publication bias was assessed using funnel plots for visual inspection and Egger's test and Begg's test for binary and continuous variables respectively. The trim‐and‐fill method was performed by removing studies that caused asymmetry and filling missed studies in the funnel plot. Sensitivity analysis was performed by dropping one study at a time to check if the findings will remain the same.

### 2.7 Certainty Assessment

Quality of evidence assessment was performed using GRADE to generate the confidence on the correctness of estimate of effect for each outcome. Since included studies were RCTs, the initial rating of the quality of evidence of each outcome was high. Downgrading was done based on risk of bias, heterogeneity, imprecision, indirectness and publication bias.

## 3 Results

### 3.1 Search Results

**FIGURE 1:**
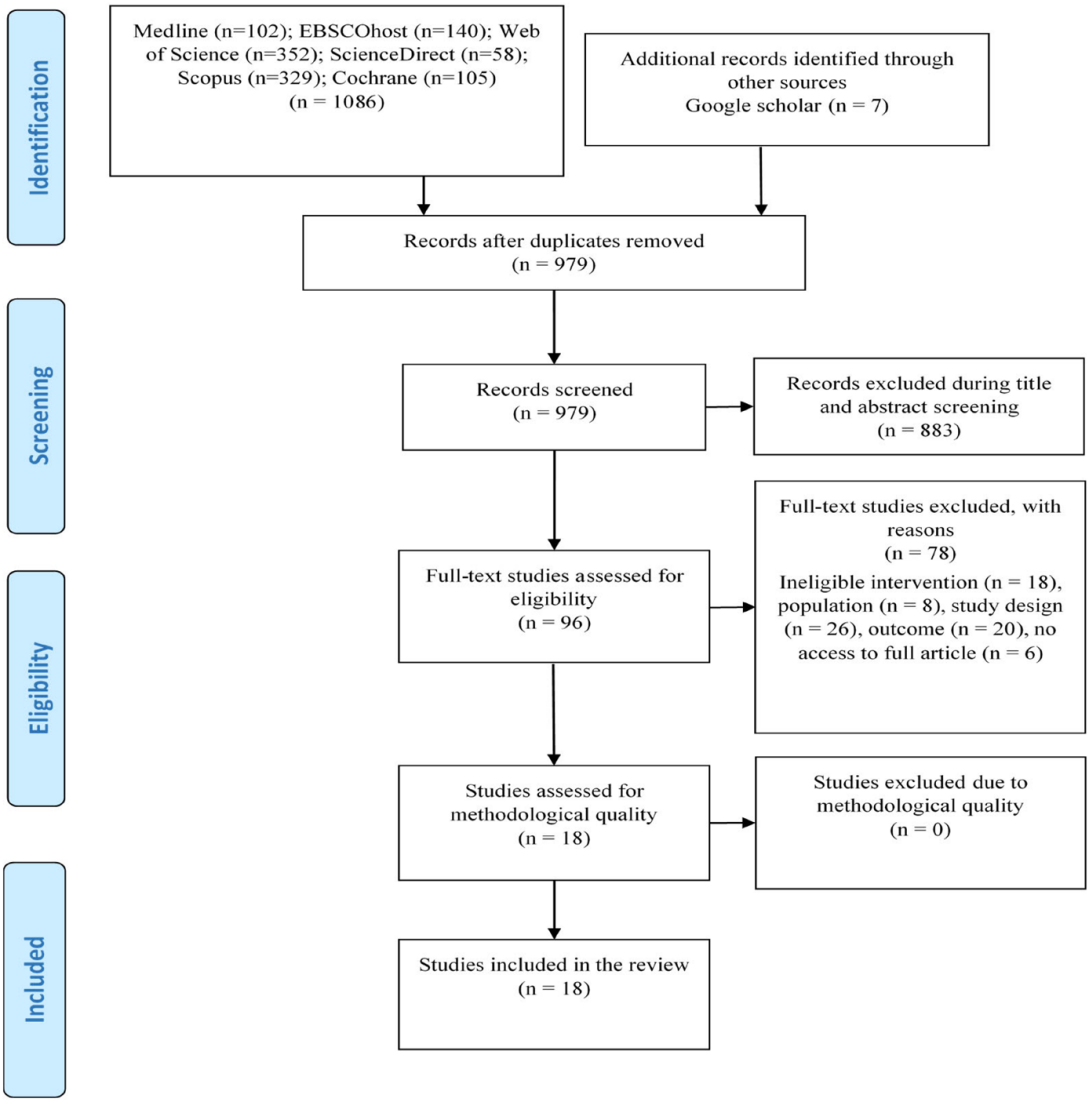
PRISMA flow diagram.

Search from the databases and other sources retrieved 1093 studies. The first reviewer removed all duplicates and left 979 studies that were available for the abstract and title screening. Then, 883 studies were removed after the abstract and title screening, leaving a total of 96 studies that were accessible for eligibility screening of full texts by two independent reviewers, until consensus was reached. Only 18 studies (Figure 1) met the eligibility criteria, whereas 78 studies were excluded following ineligible population (8 studies), intervention of interest (18 studies), outcome (20 studies), research design (26 studies), and no access to full article (6 studies). The excluded studies are detailed in Table S2 with accompanying reasons for exclusion.

### 3.2 Study Characteristics

A total of 18 studies (Table 1) published from 2002 to 2023 were included (Allen et al. 2009, 2002; Askim et al. 2004; Boter 2004; Burton and Gibbon 2005; Chalermwannapong et al. 2010; Chen et al. 2018; Chu et al. 2020; Yasemin and Gözüm 2023; Duncan et al. 2020; Fjaertoft et al. 2004; Wong et al. 2022; Lin et al. 2022; Liu 2018; Mayo et al. 2008; Mohammadi, Hassandoost, and Mozhdehipanah 2022; Mou, Lam, and Chien 2023; Wong and Yeung 2015) in this review. The total number of stroke survivors in the selected studies was 8670 with a range from 40 to 5882 stroke survivors per study. The included studies evaluated the effects of nurse‐led TC programs on quality of life (*n* = 16) (Allen et al. 2009, 2002; Askim et al. 2004; Boter 2004; Burton and Gibbon 2005; Chalermwannapong et al. 2010; Chu et al. 2020; Duncan et al. 2020; Fjaertoft et al. 2004; Wong et al. 2022; Lin et al. 2022; Liu 2018; Mayo et al. 2008; Mohammadi, Hassandoost, and Mozhdehipanah 2022; Mou, Lam, and Chien 2023; Wong and Yeung 2015), mortality (*n* = 11) (Allen et al. 2009, 2002; Askim et al. 2004; Boter 2004; Burton and Gibbon 2005; Chu et al. 2020; Duncan et al. 2020; Fjaertoft et al. 2004; Wong et al. 2022; Lin et al. 2022; Mayo et al. 2008), and readmission (*n* = 7) (Allen et al. 2002; Boter 2004; Chen et al. 2018; Yasemin and Gözüm 2023; Lin et al. 2022; Mayo et al. 2008; Wong and Yeung 2015). Furthermore, these studies were conducted in 9 countries: China (*n* = 7) (Chen et al. 2018; Chu et al. 2020; Wong et al. 2022; Lin et al. 2022; Liu 2018; Mou, Lam, and Chien 2023; Wong and Yeung 2015), United States (*n* = 3) (Allen et al. 2009, 2002; Duncan et al. 2020), Norway (*n* = 2) (Askim et al. 2004; Fjaertoft et al. 2004), Iran (*n* = 1) (Mohammadi, Hassandoost, and Mozhdehipanah 2022), Thailand (*n* = 1) (Chalermwannapong et al. 2010), Netherlands (*n* = 1) (Boter 2004), Canada (*n* = 1) (Mayo et al. 2008), Turkey (*n* = 1) (Yasemin and Gözüm 2023), and United Kingdom (*n* = 1) (Burton and Gibbon 2005).

**TABLE 1:** Characteristics of the included studies.

Authors' names, year, setting	Nature of participants	Transition care interventions	Guiding framework	Control group	Intervention group	Intensity of interventions	Outcomes and measurements	Findings
Allen et al. 2009; USA	Total = 389; IG = 190 and CG = 190. Mean age = 68.5; IG Mean = 68 and CG Mean = 69. Sex (male) = 50%; IG = 48%; and CG = 52%	Discharge planning, medication reconciliation, care coordination, collaboration, follow‐up phone calls, education and counselling	Recommendations from the national stroke association, american heart association and the royal college of physicians stroke clinical guidelines	Usual post discharge care plus reminder emails and stroke‐related educational materials	Discharge assessment followed by nurse‐led home visit, telephone calls, stroke education and personal health record	Four times in a week during hospitalisation, then once monthly. Total duration = 6 months	Quality of life: Stroke Specific Quality of life Mortality	No effect on post‐discharge quality of life (*p* = 0.53)
Allen et al. 2002; USA	Total = 93; IG = 47 and CG = 46. Mean age = 70.5; IG Mean = 69, and CG Mean = 72. Sex (male) = 45%; IG = 43%; and CG = 47%	Discharge planning, medication reconciliation, care coordination, collaboration, follow‐up phone calls, education and counselling	Recommendations from the national stroke association, american heart association and the royal college of physicians stroke clinical guidelines	Usual post discharge care plus nurse in‐home assessment at the end of the 3‐month study period	Discharge assessment followed by nurse‐led home visit, telephone calls, stroke education and personal health record.	3–7 days post discharge phone call followed with home visit in 1 month. Total duration = 3 months	Quality of Life: Sickness Impact Profile version Mortality Readmission	Improved post‐discharge quality of life (*p* = 0.0001)
Askim et al. 2004; Norway	Total = 62; IG = 31 and CG = 31. Mean age = 76.6; IG Mean = 76.9 and CG Mean = 76.3. Sex (male) = 46.9%; IG = 48.4 and CG = 45.2%	Early supported discharge, home‐based rehabilitation, collaboration with primary healthcare system, follow‐up care and care co‐ordination	Early supported discharge and coordination of rehabilitation	Usual stroke unit rehabilitation	Stroke unit rehabilitation, pre‐discharge family meeting and discharge planning, followed by home visit and telephone calls as well as local support group meeting	Stroke unit rehabilitation and 4 weeks post discharge. Total duration = 1 month	Quality of Life: Nottingham Health Profile Mortality	No effect on post discharge quality of life (*p* = 0. 918)
Boter 2004; Netherlands	Total = 536; IG = 263 and CG = 273. Mean age = 60.2; IG median and IQR = 66, 52–76 and CG median and IQR = 63, 51–74. Sex (male): 48.5% IG:49% and CG:48%	Education, problem solving, counselling and signposting	Outreach nurse support after stroke	Usual stroke unit care	Standard stroke care plus post‐discharge stroke risk factors education and assessment, unmet needs assessment, problem‐solving approach, referral to physicians	3 telephone calls and 1 home visit. Total duration = 6 months	Quality of life: Short Form‐36 Readmission Mortality	Improved post‐discharge quality of life (95% CI = 0.1 to 15.7)
Burton and Gibbon 2005; UK	Total = 176; IG = 87 and CG = 89. Mean age = 75. 25; IG = 75.8 and CG = 74.7. Sex (male) = 46%; IG = 47% and CG = 45%	Discharge planning, follow‐up visit, task training, medication reconciliation, emotional management, education, support systems, care coordination	United kingdom medical research council's guidance on the evaluation of complex healthcare interventions	Usual care on discharge from the rehabilitation unit that conforms to principles of best practice	Stroke unit care and discharge planning followed by follow‐up visit from the stroke nurse	1–28 contacts. Total duration = 32 months	Quality of Life: Nottingham Health Profile Mortality	Improved post‐discharge quality of life (*p* = 0.012)
Chalermwannapong et al. 2010; Thailand	Total = 67; IG = 33 and CG = 34. Mean age = 65.35; IG = 60.15 ± 10.23 and CG = 60.24 ± 11.13. Sex (male):53%; IG = 48%; and CG = 58%	Discharge planning, education, social support, counselling, care coordination, task training, information exchange, medication reconciliation, home visit	Naylor transitional care model	Usual care done	In‐hospitalisation baseline assessment, stroke education, skills training, medication reconciliation, stress management and home visits	Daily hospital visit, 2 home visits and 2 times telephone calls. Total duration = 4 weeks	Quality of Life: Ferrans and Powers' Quality of Life Index‐Stroke Version	Improved post‐discharge quality of life (*p* < 0.05)
Chen et al. 2018; China	Total = 144; IG = 72 and CG = 72. Mean age = 75.22; IG = 65.92 ± 12.8 and CG = 64.78 ± 9.87. Sex (male): 73.5% IG:72% and CG:75%	Education, goal setting, problem solving, surveillance and positive reinforcement	Health empowerment theory	Usual nursing care plus unstructured health education and social phone calls	Pre‐discharge needs assessment, stroke education, problem‐solving, self‐monitoring skills, rehabilitation, goal setting, reinforcements and empowerment	One small group session, 1 discharge session, and 4 weekly calls. Total duration = 6 weeks	Readmission	Reduced rates of readmission at both 1 and 3 months
Chu et al. 2020; China	Total = 61; IG = 31 and CG = 30. Mean age = 64.5 ± 9.2, range = 43 79. Sex (male) = 39.3%	Rehabilitation skills and follow‐up	Not reported	Conventional care	Training caregivers	Once per day for 60 min, a total of 3 times. Total duration = 4 weeks	Quality of Life: EuroQoL‐5Dimension Mortality	No effect on post‐discharge quality of life (*p* = 0. 91)
Duncan et al. 2020; USA	Total = 5882; IG = 2689 and CG = 3193. Mean age IG = 68.0 ± 13.8 and CG = 66.3 ± 13.9. Sex (male) = 49.9%; IG 51.7% and CG = 48.1%	Follow‐up, discharge planning, education, rehabilitation, collaboration, emotional and risk behaviours management, medication reconciliation	Transitional care model for stroke	Current standard of post‐acute care	Standard post‐acute care, telephone follow‐up and a clinic visit	Total duration = 3 months	Quality of Life: Stroke Impact Scale‐16 Survival at 90 days	No on post‐discharge quality of life (95% CI = −1.74 to 2.97)
Fjaertoft et al. 2004; Norway	Total = 320; IG = 160 and CG = 160. Age and sex: not reported	Early supported discharge, rehabilitation, home visits, care coordination and collaboration	Early supported discharge and coordination of rehabilitation	In‐hospital stroke unit rehabilitation	Stroke unit rehabilitation, pre‐discharge meetings, home visit and home‐based rehabilitation	Total duration = 3 months.	Quality of Life: Nottingham Health Profile Mortality	Improved post‐discharge quality of life (*p* = 0.048)
Lin et al. 2022; China	Total = 140; IG = 70 and CG = 70. Total = 140; IG = 70 CG = 70. Mean age = 61.7. Sex (male) = 75.7%; IG = 77.1% and CG = 74.3%	Goal setting, education, task training, medication management; and follow‐up	Self‐efficacy theory	Usual transitional discharge plan	Individualised coaching sessions prior to discharge followed by telephone support and face‐to‐face coaching activities	Weekly telephone calls and bi‐weekly face‐to‐face coaching. Total duration = 12 weeks	Quality of Life: Stroke‐Specific Quality of Life Scale Readmission Mortality	Improved post‐discharge quality of life (*p* < 0.001)
Liu 2018; China	Total = 40; IG = 20 and CG = 20. Mean age = 69.5; IG = 76.32 ± 4.56 and CG = 74.12 ± 3.12. Sex (male):60%; IG:55% and CG:65%	Discharge planning, education, surveillance, information exchange, exercise training, home visits, phone calls and medication adjustments	Naylor transitional care model	Routine care	Individualised assessment coupled with comprehensive education, rehabilitation and follow‐up visits	Total duration = 2 months	Quality of Life: Short Form‐36	Improved post‐discharge quality of life (*p* < 0.01)
Mayo et al. 2008; Canada	Total = 190; IG = 96 and CG = 94. Mean age = 66; IG = 70 ± 14.5 and CG = 72 ± 12.95 Sex (male): 61% IG:67%; and CG:55%	Care coordination, education, signposting, counselling, information exchange, surveillance and caregiver support	Case‐management model	Usual care plus instructed follow‐up appointment	Customised discharge summaries, follow‐up visit, surveillance, information exchange, medication reconciliation, family support, and education	Total duration = 6 weeks	Quality of Life: Short Form‐36 and EuroQoL‐5‐Dimension Readmission Mortality	No effect on post‐discharge quality of life hospital readmissions
Mohammadi, Hassandoost, and Mozhdehipanah 2022; Iran	Total = 67; IG = 31 and CG = 36. Mean age = 68.21; IG 66.80 ± 6.05 and CG = 69.61 ± 8.36. Sex (male) = 64.5%; IG = 65% and CG = 64%	Education, partnership training, follow‐up, diet and activity modification, medication reconciliation, information exchange	Partnership care model	Routine care at the rehabilitation center	In‐hospital educational sessions plus post‐discharge follow‐up visits (sessions)	Three weekly sessions and monthly follow‐up. Total duration = 6 months	Quality of Life: Stroke‐Specific Quality of Life Scale	Improved post‐discharge quality of life (*p* < 0.05)
Mou, Lam, and Chien 2023; China	Total = 162; IG =81 and CG = 81. Mean age = 56.07 ± 11.17); IG = 54.63 ± 11.80 and CG = 57.52 ± 10.37. Sex (male) = 75.7; IG = 77.1 and CG = 74.3	Dyadic psychoeducation, follow‐up after discharge and phone calls	Double ABC‐X model	Usual care plus 1–2 post‐stroke lessons	In‐hospital education sessions and post‐hospitalisation phone calls	Total duration = 4 weeks	Quality of Life: Stroke Impact Scale	Improved post‐discharge quality of life (*p* = 0.025)
Wong and Yeung 2015; China	Total = 108; IG = 54 and CG = 54. Mean age = 64.5; IG = 67.5 ± 11.6 and CG = 71.5 ± 11.6. Sex (male):32%; IG:27% and CG:37%	Home‐based exercise, surveillance, education, counselling, care coordination, self‐ management, medication reconciliation and social support	Omaha system	Routine care	Pre‐discharge family meeting coupled with home visits, task training, emotional management, health education, counselling, information exchange, social support and self‐monitoring	One pre‐discharge family meeting and 4× weekly home visits. Total duration = 4 weeks	Quality of Life: Short Form‐36 & WHO‐Quality of Life Readmission	Improved post‐discharge quality of life (*p* = 0.005)
Wong et al. 2022; China	Total = 116; IG = 58 and CG = 58. Mean age = 66.6 ± 9.3; IG = 66.21 ± 10.07) and CG = 67.00 ± 8.61. Sex (male) = 69.8; IG = 72.4% and CG = 67.2%	Education, counselling, surveillance, case management, problem assessment, goal setting, task training, phone calls, home‐based exercise, follow‐up, coordination and collaboration	4C Transitional care framework with omaha system	Standard care	Pre‐discharge meeting, routine post‐discharge advice, task training, home‐based follow‐up in accordance with the omaha System	One pre‐discharge session, 6 post‐discharge home visits and 6 telephone follow‐up calls. Total duration = 12 weeks	Quality of Life: EuroQoL‐5‐Dimension and Stroke Impact Scale Mortality	Improved post‐discharge quality of life (*p* < 0.001)
Yasemin and Gözum 2023; Turkey	Total = 126; IG =66 and CG = 60. Mean age IG = 60.97 ± 15.73 and CG = 62.09 ± 13.72. Sex (male) = 52.2%; IG = 57.6% and CG = 46.7%	Follow‐up after discharge, hospital visits, home visit, phone calls, and Web‐based training	Transitional care Model	Routine hospital discharge	Pre‐discharge education, follow‐up phone calls, home visits and WHATSAPP calls and texts	Total duration = 12‐weeks	Readmission	Reduced rates of readmission

Abbreviations: CG, control (usual care) group; IG, intervention (transition care) group; QoL, quality of life; WHO, world health organisation.

Only 3 studies reported patient stroke severity using the National Institutes of Health Stroke Scale (NIHSS) (Allen et al. 2009, 2002; Duncan et al. 2020). Majority of the participants were males (53.3%) with the mean age between 56.07 and 76.6. Included participants had the diagnosis of ischemic stroke or hemorrhagic stroke. Except two studies, other studies reported the support systems for stroke survivors such as care givers, family aides or helpers, spouse, children, local agencies, local healthcare system and caregiver support services (Allen et al. 2009; Boter 2004; Burton and Gibbon 2005; Chalermwannapong et al. 2010; Chen et al. 2018; Chu et al. 2020; Yasemin and Gözüm 2023; Duncan et al. 2020; Fjaertoft et al. 2004; Wong et al. 2022; Lin et al. 2022; Liu 2018; Mayo et al. 2008; Mohammadi, Hassandoost, and Mozhdehipanah 2022; Mou, Lam, and Chien 2023; Wong and Yeung 2015). In all studies, participants were recruited from the hospitals whereby they received TC led by skilled nurses. Participants in TC group received either home visits or telephone calls by TC nurses.

### 3.3 Transition Care Intervention Characteristics

Except for only one study (Chu et al. 2020), other studies used theoretical frameworks, and national and international guideline recommendations to develop nurse‐led TC interventions (Table 1). The common TC interventions were discharge planning; bi‐directional information exchange; medication reconciliation; counselling and education on risk reduction and complication avoidance; collaboration with multidisciplinary team and primary healthcare providers; follow‐up home visits or phone calls; care coordination; symptom management and monitoring; enlisting social support from community‐based resources; and problem‐solving skills and rehabilitation training. The TC interventions were delivered through face‐to‐face interactions, online platforms or a blended mode.

### 3.4 Transition Care Program Delivery

Stroke survivors in the control group received discharge and post‐hospitalisation care according to the routine program of the hospitals. The usual care included general nursing care, hospital booking appointments, medication advice and routine rehabilitation sessions. Further, participants in the control groups received verbal health education, routine family care, and social phone calls (Table 1).

Whereas, stroke survivors in the intervention group received usual care plus nurse‐led TC program interventions. Additionally, during hospitalisation, the stroke survivors participated in discharge planning and discharge readiness assessment. Stroke survivors received in‐hospital education, counselling and skill training. Prior discharge, TC nurses organised meetings with patients and their families to discuss about home visits or follow‐up calls. At discharge, stroke survivors were given brochures that contain customised stroke‐related education contents for self‐review. Also, stroke survivors were given personalised mini‐health records that were shared with their primary care physicians. After discharge, the TC nurses followed the stroke survivors at home or made a phone calls.

### 3.5 Risk of Bias of the Included Studies

Based on the JBI critical appraisal tool for randomised controlled trials, all 18 included studies had a true randomization process for participants' allocation (Table 2). However, 5 studies did not employ clear concealment allocation strategies (Chalermwannapong et al. 2010; Chu et al. 2020; Yasemin and Gözüm 2023; Fjaertoft et al. 2004; Liu 2018). In all the 18 studies, there was no statistically significant difference in baseline characteristics between intervention and control groups. Similarly, in all 18 studies, nurse implementers were not blinded on group allocations. In 2 studies (Liu 2018; Mohammadi, Hassandoost, and Mozhdehipanah 2022), outcome assessors were not blinded on group allocations. Only 2 studies (Boter 2004; Mohammadi, Hassandoost, and Mozhdehipanah 2022), blinded the participants on group allocations. All 18 studies adhered to intervention protocol with no additional treatment offered in a group. Except only 1 study (Liu 2018), other studies provided reasons for participants' loss of follow‐up. Likewise, except 2 studies (Allen et al. 2002; Mohammadi, Hassandoost, and Mozhdehipanah 2022), other studies employed intention‐to‐treat principle analysis to ascertain for TC program effectiveness. Finally, all studies used validated measurement tools and appropriate study RCT design. Nonetheless, majority of studies (*n* = 11) had an attrition rate of < 5% (Allen et al. 2009, 2002; Boter 2004; Burton and Gibbon 2005; Fjaertoft et al. 2004; Wong et al. 2022; Lin et al. 2022; Mayo et al. 2008; Mohammadi, Hassandoost, and Mozhdehipanah 2022; Mou, Lam, and Chien 2023; Wong and Yeung 2015).

**TABLE 2:** Risk of bias for included studies.

Authors' names and year	Q1	Q2	Q3	Q4	Q5	Q6	Q7	Q8	Q9	Q10	Q11	Q12	Q13	TOTAL
Allen et al. 2009	Y	Y	Y	U	N	Y	Y	Y	Y	Y	Y	Y	Y	11
Allen et al. 2002	Y	Y	Y	U	N	Y	Y	Y	U	Y	Y	Y	Y	10
Askim et al. 2004	Y	Y	Y	U	N	Y	Y	Y	Y	Y	Y	Y	Y	11
Boter 2004	Y	Y	Y	Y	U	Y	Y	Y	Y	Y	Y	Y	Y	12
Burton and Gibbon 2005	Y	Y	Y	N	N	Y	Y	Y	Y	Y	Y	Y	Y	11
Chalermwannapong et al. 2010	Y	U	Y	U	U	Y	Y	Y	Y	Y	Y	Y	Y	10
Chen et al. 2018	Y	Y	Y	U	N	Y	Y	Y	Y	Y	Y	Y	Y	11
Chu et al. 2020	Y	U	Y	U	N	Y	Y	Y	Y	Y	Y	Y	Y	10
Duncan et al. 2020	Y	Y	Y	U	U	Y	Y	Y	Y	Y	Y	Y	Y	11
Fjaertoft et al. 2004	Y	U	Y	U	U	Y	Y	Y	Y	Y	Y	Y	Y	10
Lin et al. 2022	Y	Y	Y	U	U	Y	Y	Y	Y	Y	Y	Y	Y	11
Liu 2018	Y	U	Y	U	U	U	Y	U	Y	Y	Y	Y	Y	8
Mayo et al. 2008	Y	Y	Y	U	U	Y	Y	Y	Y	Y	Y	Y	Y	11
Mohammadi, Hassandoost, and Mozhdehipanah 2022	Y	Y	Y	Y	N	N	Y	Y	N	Y	Y	Y	Y	10
Mou, Lam, and Chien 2023	Y	Y	Y	N	N	Y	Y	Y	Y	Y	Y	Y	Y	11
Wong and Yeung 2015	Y	Y	Y	U	U	Y	Y	Y	Y	Y	Y	Y	Y	11
Wong et al. 2022	Y	Y	Y	U	U	Y	Y	Y	Y	Y	Y	Y	Y	11
Yasemin and Gözum 2023	Y	U	Y	N	N	Y	Y	Y	Y	Y	Y	Y	Y	10

*Note:* Q1 to Q13: question numbers according to JBI critical appraisal tool for randomised controlled trials.

Abbreviations: N, no; U, unclear; Y, yes.

### 3.6 Effects of Nurse‐Led TC Programs on Post‐Discharge Outcomes

#### 3.6.1 Effects of Nurse‐Led TC Programs on Quality of Life

**FIGURE 2:**
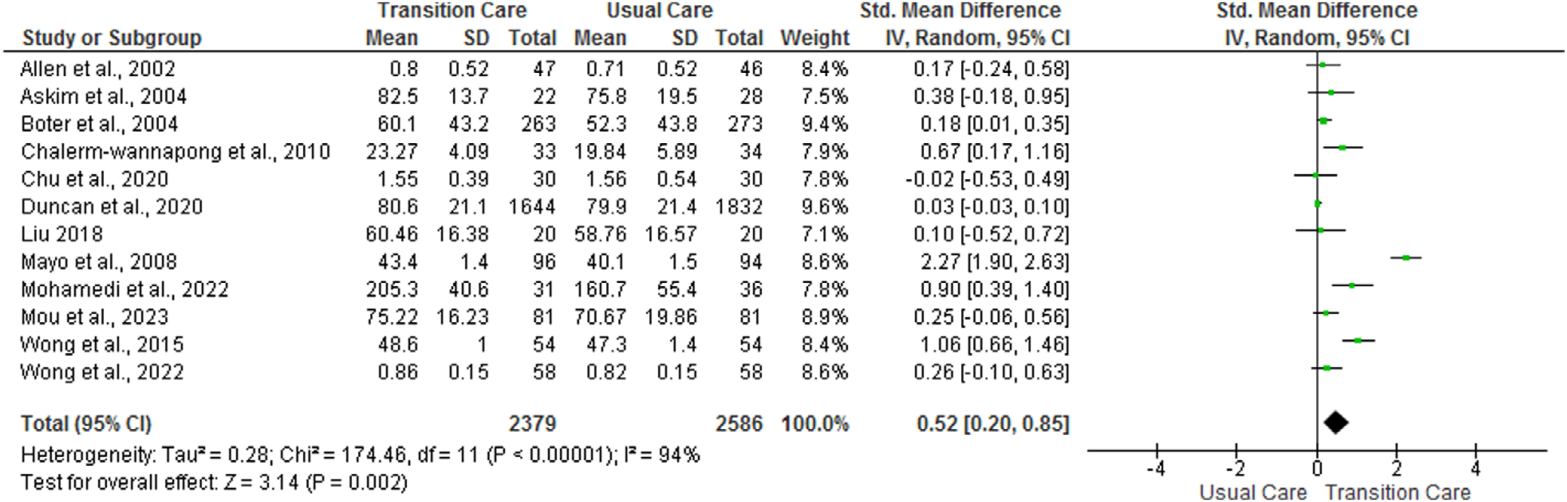
The pooled effect of nurse‐led transition care programs on stroke survivors' quality of life.

Out of 18 studies reviewed, 16 studies reported the effect of nurse‐led TC programs on quality of life. Quality of life was measured by 8 different tools. The common tools were Stroke Specific Quality of Life (Allen et al. 2009; Lin et al. 2022; Mohammadi, Hassandoost, and Mozhdehipanah 2022), Stroke Impact Scale (Duncan et al. 2020; Mou, Lam, and Chien 2023), Short Form‐36 (Boter 2004; Liu 2018; Mayo et al. 2008; Wong and Yeung 2015), Euro‐Quality of life‐5‐Level, 5‐Dimension (Chu et al. 2020; Wong et al. 2022) and Nottingham Health Profile (Burton and Gibbon 2005; Fjaertoft et al. 2004). Of 16 studies, 11 studies reported statistically significant positive effect (*p* < 0.05) within 1‐year of stroke (Allen et al. 2002; Boter 2004; Burton and Gibbon 2005; Chalermwannapong et al. 2010; Chen et al. 2018; Yasemin and Gözüm 2023; Fjaertoft et al. 2004; Wong et al. 2022; Lin et al. 2022; Liu 2018; Mohammadi, Hassandoost, and Mozhdehipanah 2022; Mou, Lam, and Chien 2023; Wong and Yeung 2015). A short‐term (< 6 months) positive effect (*p* < 0.05) was reported in 9 studies (Allen et al. 2002; Burton and Gibbon 2005; Chalermwannapong et al. 2010; Wong et al. 2022; Lin et al. 2022; Liu 2018; Mohammadi, Hassandoost, and Mozhdehipanah 2022; Mou, Lam, and Chien 2023; Wong and Yeung 2015), whereas the medium‐term (6 months to 1 year) positive effect (*p* < 0.05) was reported in 6 studies (Boter 2004; Burton and Gibbon 2005; Fjaertoft et al. 2004; Wong et al. 2022; Lin et al. 2022; Mohammadi, Hassandoost, and Mozhdehipanah 2022). Four studies (Allen et al. 2009; Burton and Gibbon 2005; Fjaertoft et al. 2004; Lin et al. 2022), were not pooled in meta‐analysis because they lacked appropriate effect size estimates. Results from the meta‐analysis (Figure 2) show the statistically significant positive medium effect of nurse‐led TC programs compared with usual care (SMD = 0.52; 95% CI = 0.20, 0.85; *p* = 0.002; *I* ^2^ = 94%). Subgroup analysis (Figure S1) revealed positive large effect of nurse‐led TC programs compared with usual care both at less than 6 months (SMD = 1.11; 95% CI = 0.11, 2.12; *p* = 0.03; *I* ^2^ = 87%), and from 6 months to 1 year (SMD = 1.55; 95% CI = 0.22, 2.89; *p* = 0.03; *I* ^2^ = 98%).

#### 3.6.2 Effects of Nurse‐Led TC Programs on Mortality

**FIGURE 3:**
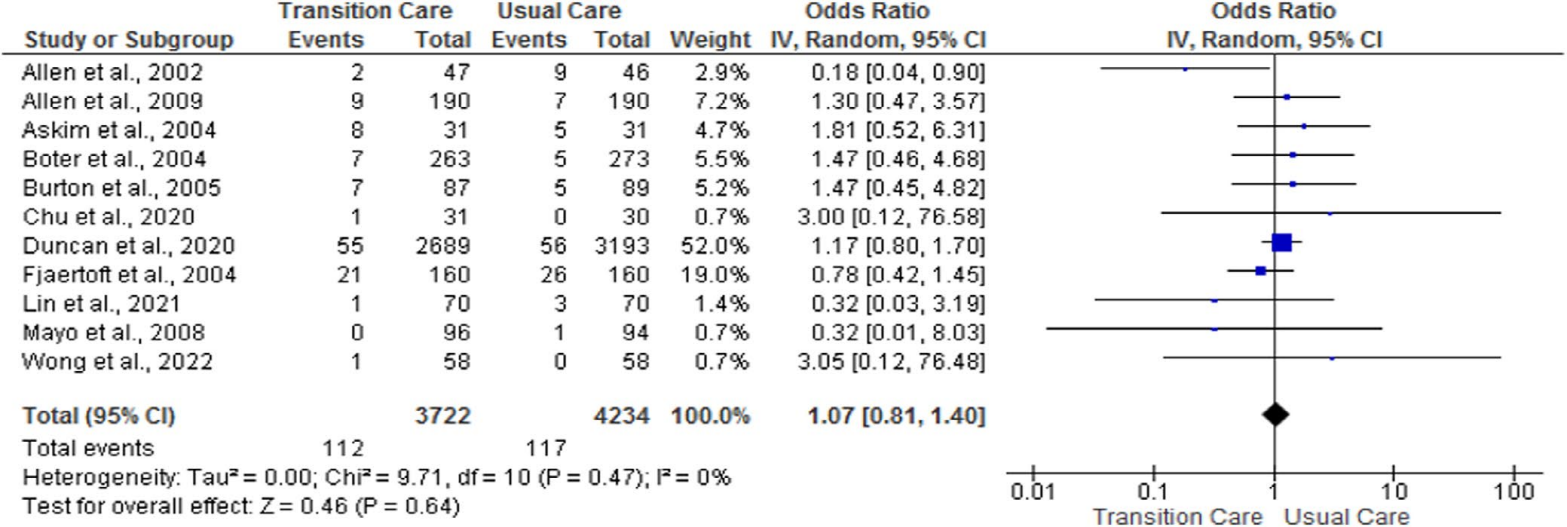
The pooled effect of nurse‐led transition care programs on mortality.

Eleven out of 18 studies reported the effect of nurse‐led TC programs on mortality (Allen et al. 2009, 2002; Askim et al. 2004; Boter 2004; Burton and Gibbon 2005; Chu et al. 2020; Duncan et al. 2020; Fjaertoft et al. 2004; Wong et al. 2022; Lin et al. 2022; Mayo et al. 2008). The pooled data from the meta‐analysis (Figure 3) however show no statistically significant effect of nurse‐led TC programs compared with usual care (OR = 1.07; 95% CI = 0.81, 1.40; *p* = 0.64; *I* ^2^ = 0%) at 1 year. Subgroup analysis (Figure S2) didn't show any statistically significant effect of nurse‐led TC programs compared with usual care at less than 6 months (OR = 0.73; 95% CI = 0.18, 3.00; *p* = 0.66; *I* ^2^ = 63%) and from 6 months to 1 year (OR = 1.06; 95% CI = 0.70, 1.59; *p* = 0.79; *I* ^2^ = 0%).

#### 3.6.3 Effects of Nurse‐Led TC Programs on Readmissions

**FIGURE 4:**
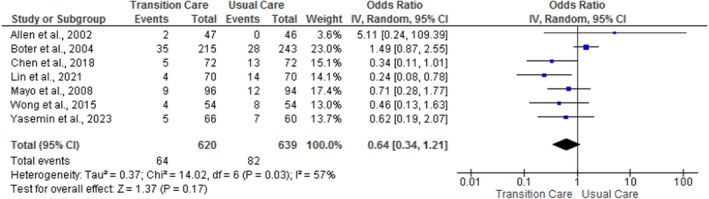
The pooled effect of nurse‐led transition care programs on readmission.

Among 18 studies, 7 studies reported the effects of nurse‐led TC programs on readmission (Allen et al. 2002; Boter 2004; Chen et al. 2018; Yasemin and Gözüm 2023; Lin et al. 2022; Mayo et al. 2008; Wong and Yeung 2015). The pooled data from the meta‐analysis (Figure 4) show no statistically significant effect of nurse‐led TC programs compared with usual care (OR = 0.64; 95% CI = 0.34, 1.21; *p* = 0.17; *I* ^2^ = 57%) at 1 year. Subgroup analysis (Figure S3) show a statistically significant medium effect of nurse‐led TC programs compared with usual care at less than 6 months (OR = 0.50; 95% CI = 0.26, 0.98; *p* = 0.04; *I* ^2^ = 0%) but not from 6 months to 1 year (OR = 0.71; 95% CI = 0.26, 1.93; *p* = 0.50; *I* ^2^ = 76%).

### 3.7 Publication Bias

The funnel plots show asymmetrical distribution of the studies (Figures S4a–S4c) and the results of Begg's and Egger's tests suggested that there was significant evidence of publication bias for all outcomes (*p* > 0.05). The trim‐and‐fill method did not estimate possible missing studies in all outcomes (Figures S5a–S5c).

### 3.8 Sensitivity Analysis

After removing one trial at a time, there was evidence of change in the effect size in readmission but not in quality of life and mortality. The change ranged from OR = 0.64; 95% CI = 0.34, 1.21; *p* = 0.17 to OR = 0.72; 95% CI = 0.23, 1.21; *p* = 0.004 (Figures S6a–S6c). This shows that an individual study had a significant influence on the overall result of readmission but not quality of life and mortality.

### 3.9 Certainty of Evidence

For the outcome quality of life, the overall quality of evidence was downgraded two levels because of high heterogeneity, and indirectness of evidence because of inconsistency in the model components and training of nurse implementers. For the outcome mortality, the overall quality of evidence was downgraded two levels because of indirectness of evidence because of inconsistency in the model components and publication bias; whereas, for the outcome readmission, the overall quality of evidence was downgraded three levels because of heterogeneity, indirectness of evidence because of inconsistency in the model components and publication bias. Therefore, the overall quality of evidence for quality of life and readmission was low, while that of readmission was very low (Table 3).

**TABLE 3:** Summary of findings on the effectiveness of nurse‐led TC programs for stroke survivors population: adult stroke patients setting: hospital‐ to‐home transition intervention: nurse‐led transition care comparison: usual transition care.

Outcomes	Illustrative comparative risk		Overall effects (95% CI)	No. participants (studies)	Quality of evidence (GRADE)	Comments
	Usual TC	Nurse‐led TC				
Quality of life	See comment	See comment	SMD = 0.52 (0.20, 0.85)	4965 (12)	Low	Higher score indicates better quality of life
Mortality	117 out of 4234 died	112 out of 3722 died	OR = 1.07 (0.81, 1.40)	7956 (11)	Low	229 participants died in 11 studies
Readmission	82 out of 639 readmitted	64 out of 620 readmitted	OR = 0.64 (0.34, 1.21)	1259 (7)	Very low	146 participants were readmitted in 7 studies

## 4 Discussion

This study found a significant medium effect of nurse‐led TC programs on stroke survivors' quality of life but not on the odds of reducing mortality and readmission at 1 year. Previous studies on nurse‐led TC have shown that these programs play a crucial role in improving stroke survivors' quality of life and readmissions (Deepradit et al. 2023; Condon et al. 2016). But a previous meta‐analysis has found limited evidence to support the effectiveness of TC in improving quality of life, reducing mortality and decreasing readmissions among stroke survivors (Puhr and Thompson 2015). The observed incongruences may be due to differences in patient demographics, methodological quality and healthcare settings.

Some of nurse‐led TC programs have been focusing on elderly stroke population (Askim et al. 2004; Boter 2004; Burton and Gibbon 2005). Old age is associated with cognitive impairment and care dependency that might mask the effectiveness of TC programs. Similarly, the methodological inequalities and ceiling effect can affect the effectiveness of nurse‐led TC programs (Jee et al. 2022). Thus, assessing the effectiveness of TC programs requires robust measurement and evaluation tools across multiple domains. In our study, eight tools with different psychometric properties were used to measure quality of life. Such variation in assessment tools may pose challenges in identifying the true effect of TC programs. Therefore, validated tools and other relevant indicators such as activities of daily living, should be used to understand the holistic impact of nurse‐led TC programs (Jee et al. 2022).

Similarly, healthcare system characteristics and implementation strategies can affect the effectiveness of nurse‐led TC programs (Fakha et al. 2021). For example, healthcare systems with robust primary care infrastructure and community‐based support services may be better positioned to implement nurse‐led TC programs and achieve positive outcomes. However, in regions facing severe nursing staff shortages, implementing nurse‐led TC programs may be problematic (Lutz et al. 2020). TC programs require well‐trained and deployed nurses to coordinate its implementation. Nevertheless, in areas facing a critical nursing staffing shortage, available nurses maybe trained to train family caregivers to help in delivering some TC components.

Involving family caregivers can help to tailor nurse‐led TC interventions to meet individual needs. Developing personalised and culturally sensitive TC programs that address the specific needs and preferences of stroke survivors can enhance models' effectiveness and relevance (Bushnell et al. 2018; Vadas et al. 2022). Equally, nurses should employ health literacy principles and provide comprehensive support to caregivers. Empowering caregivers with knowledge, skills, and resources can reduce caregiver burden and enhance coping abilities, leading to better post‐discharge outcomes for both caregivers and stroke survivors (Cook and Pompon 2023).

Engaging community resources, support networks and peer‐led programs can complement nurse‐led TC interventions. Peer support groups provide additional layers of support for stroke survivors and their caregivers (Christensen, Golden, and Gesell 2019). Collaborating with community organisations, faith‐based groups, and volunteer networks can expand access to social services and other support services that promote holistic recovery (Oshvandi et al. 2024). Building strong partnerships with community stakeholders may foster a sense of belonging, social connectedness, and resilience among stroke survivors. Such connections are essential for the long‐term recovery and overall quality of life of stroke survivors.

Leveraging technology solutions, such as telehealth, mobile apps, and remote monitoring devices, can expand access and effectiveness of nurse‐led TC programs (Hwang, Park, and Chang 2021). Integrating technology into nurse‐led TC interventions offers opportunities to enhance symptom monitoring and self‐management support for stroke survivors. In our current review, some TC programs used websites and social networking platforms to render TC interventions. Telehealth platforms can facilitate virtual consultation clinics, symptom tracking, medication reconciliation and rehabilitation adherence. However, challenges such as access barriers, digital literacy, and privacy concerns must be addressed to ensure equitable and effective use of technology‐enabled nurse‐led TC programs (Kamoen et al. 2020).

Furthermore, to ensure effective delivery of nurse‐led TC, nurses should receive adequate training in stroke care pathways (Jarva et al. 2021). We noted that TC nurses underwent specialised training prior to delivering TC interventions. Such continuing education programs, interdisciplinary workshops, simulation‐based trainings can enhance stroke unit nurses' knowledge, skills and confidence in caring for stroke survivors during transition (Rababah 2021). Additionally, TC programs need good coordination and collaboration among healthcare providers. Promoting a culture of collaboration and shared decision‐making can foster a supportive and cohesive care environment that optimises patient outcomes across the care continuum (Chiu et al. 2021).

## 5 Limitations

The included studies used 8 measurement tools to evaluate the effectiveness of nurse‐led TC programs on quality of life that. Use of different measurement tools may have led to observed high heterogeneity between studies. Second, many studies didn't report the severity and confirmation of stroke that may have caused possibility of including patients with stroke mimics. We employed random effects models and subgroup analysis to address potential heterogeneity across studies. However, the heterogeneity remained high and possibly couldn't be resolved solely by statistical methods. Similarly, no single study was conducted in Africa, hence limiting the generalisation of the findings in African continent.

## 6 Implications for Practice, Policy and Research

This study has several implications for practices. Firstly, nurse‐led TC interventions may be integrated into comprehensive stroke care pathways to ensure continuity of care. Secondly, nurses should receive specialised training to effectively deliver stroke transition care (Zhao et al. 2024). Thirdly, interdisciplinary collaboration among healthcare providers is essential for addressing the unique needs of stroke survivors during the transition period. For policy, healthcare organisations should invest in quality improvement initiatives to support the implementation and sustainability of nurse‐led TC programs.

Future research in stroke transition care should focus on evaluating the long‐term effects, scalability and cost‐effectiveness of nurse‐led TC interventions. High quality randomised controlled trials and prospective cohort studies are needed to assess the comparative effectiveness of different transition care interventions in diverse healthcare settings. Additionally, future studies should explore the mechanisms underlying the impact of nurse‐led TC programs on patient outcomes, whereas qualitative research would provide insights into the experiences and perspectives of nurses and stroke survivors regarding the delivery and receipt of nurse‐led TC programs.

## 7 Conclusion

Nurse‐led TC programs hold promise for improving quality of life of stroke survivors. However, these programs should be designed with sustainability and scalability mind‐set. To ensure long‐term impact and widespread adoption, nurses may use existing resources, infrastructure and establish partnership with community organisations and stakeholders. Moreover, it's crucial to demonstrate the cost‐effectiveness of nurse‐led TC programs. Such economic evaluations and return on investment analyses can get support from policymakers or healthcare administrators. Such support from key stakeholders may facilitate integration of nurse‐led TC interventions into standard care pathways.

## Author Contributions

**Nyagwaswa Athanas Michael:** conceptualization, data curation, data analysis and synthesis, validation, writing – original draft, reviewing, editing and approving the final draft, and agreed to be accountable for all aspects of the work. **Lilian Teddy Mselle:** conceptualization, data curation, data analysis and synthesis, validation, writing – original draft, reviewing, editing and approving the final draft, and agreed to be accountable for all aspects of the work. **Edith Mroso Tarimo:** conceptualization, data curation, data analysis and synthesis, validation, writing – original draft, reviewing, editing and approving the final draft, and agreed to be accountable for all aspects of the work. **Yingjuan Cao:** conceptualization, project administration, supervision, validation, writing – original draft, reviewing, editing and approving the final draft, and agreed to be accountable for all aspects of the work.

## Conflicts of Interest

The authors declare no conflicts of interest.

## Supporting information


Data S1.

## Data Availability

Extracted data and other relevant data are found as Supporting Information.
