# Supplementary material for: The Effectiveness of Nurse‐Led Transition Care on Post‐Discharge Outcomes of Adult Stroke Survivors: A Systematic Review and Meta‐Analysis
Source: Nurs Open. 2025 Feb 28;12(3):e70140. doi: 10.1002/nop2.70140 (PMC11871394; doi:10.1002/nop2.70140)
Supplement: Supplementary file 1 — Data S1. [file NOP2-12-e70140-s001.docx]

**Supplementary file 1 Table 1: Search Strategy**

|  | **Ovid MEDLINE(R)** |
| --- | --- |
| 1 | exp Stroke/ |
| 2 | Stroke.mp. OR cerebrovascular accident.mp. OR cerebrovascular.mp. OR CVA.mp. |
| 3 | exp cerebrovascular disorders/ OR cerebrovascular disease.mp. |
| 4 | 1 OR 2 OR 3 |
| 5 | exp continuity of patient care/ OR continuity of patient care.mp. |
| 6 | patient discharge.mp. OR exp patient discharge/ OR discharge plan*.mp. OR patient* handoff.mp. OR patient* transfer.mp. OR patient handoff.mp. OR patient transfer.mp. OR exp patient transfer/ OR aftercare.mp. OR aftercare/ OR retention of care.mp. OR follow care.mp. |
| 7 | transition* care.mp. OR exp transitional care/ OR patient transition*.mp. OR hospital to home transition.mp. OR exp hospital to home transition/ OR care transition.mp. OR transition management.mp. |
| 8 | exp patient navigation/ OR patient navigation.mp. OR patient centred care.mp. |
| 9 | exp hospitalization/ OR post hospital*.mp. OR post hospitalization.mp. OR post discharge.mp. OR post stroke.mp. OR poststroke.mp. |
| 10 | stroke rehabilitation.mp. OR exp stroke rehabilitation/ OR transition intervention.mp. |
| 11 | 5 OR 6 OR 7 OR 8 OR 9 OR 10 |
| 12 | exp nurses/ OR registered nurse.mp. OR bedside nurse.mp. OR stroke nurse.mp. OR rehabilitation nurse.mp. OR geriatric nurse.mp. OR transition nurse.mp. |
| 13 | nurse-led.mp. OR transition coordinator.mp. OR nurse coach.mp. OR nurse coordinator.mp. |
| 14 | 12 OR 13 |
| 15 | exp quality of life/ OR quality of life.mp. OR functional disability.mp. OR disability.mp. OR activities of daily living.mp. OR functional decline.mp. OR functional impairment.mp. OR cognitive decline.mp. OR cognitive impairment.mp. OR health status.mp OR sickness impact.mp |
| 16 | patient readmission/ OR readmission.mp. OR rehospitalization.mp. |
| 17 | Mortality/ OR death.mp. |
| 18 | 15 OR 16 OR 17 |
| 19 | 4 AND 11 AND 14 AND 18 = 102 |
|  | **COCHRANE LIBRARY and CENTRAL REGISTRY OF CLINICAL TRIALS on Wiley** |
| 1 | Mesh Descriptor: [stroke] explode all trees |
| 2 | stroke:ti,ab,kw OR cerebrovascular NEXT (accident OR event): ti,ab,kw |
| 3 | #1 OR #2 |
| 4 | Mesh Descriptor:[ transitional care] explode all trees |
| 5 | continuity NEXT care:ti,ab,kw OR patient NEXT care:ti,ab,kw OR transition NEXT care:ti,ab,kw OR hospital NEXT transition:ti,ab,kw OR patient NEXT transition*:ti,ab,kw OR patient NEXT handoff:ti,ab,kw OR patient NEXT transfer:ti,ab,kw OR aftercare:ti,ab,kw |
| 6 | Mesh Descriptor: [Stroke Rehabilitation] explode all trees |
| 7 | poststroke:ti,ab,kw OR post NEXT acute:ti,ab,kw |
| 8 | Mesh Descriptor:[Patient Discharge] explode all trees |
| 9 | patient NEXT discharge:ti,ab,kw OR discharge NEXT plan*:ti,ab,kw OR post NEXT discharge:ti,ab,kw OR post NEXT hospital*:ti,ab,kw OR post NEXT hospitalization:ti,ab,kw |
| 10 | #4 OR #5 OR #6 OR #7 OR #8 OR #8 |
| 11 | Mesh Descriptor: [nurse clinicians] explode all trees |
| 12 | registered NEXT nurse:ti,ab,kw OR bedside NEXT nurse:ti,ab,kw OR stroke NEXT nurse:ti,ab,kw OR rehabilitation NEXT nurse:ti,ab,kw OR geriatric NEXT nurse:ti,ab,kw OR transition NEXT nurse:ti,ab,kw OR nurse-led:ti,ab,kw OR transition NEXT coordinator:ti,ab,kw OR nurse NEXT coach:ti,ab,kw OR nurse NEXT coordinator:ti,ab,kw OR multidisciplinary:ti,ab,kw |
| 13 | #11 OR #12 |
| 14 | Mesh Descriptor: [quality of life] explode all trees |
| 15 | functional NEXT disability:ti,ab,kw OR disability:ti,ab,kw OR functional NEXT decline:ti,ab,kw OR functional NEXT impairment:ti,ab,kw OR physical NEXT function:ti,ab,kw OR cognitive NEXT decline:ti,ab,kw OR cognitive NEXT impairment:ti,ab,kw OR health NEXT status:ti,ab,kw OR sickness NEXT impact:ti,ab,kw |
| 16 | Mesh Descriptor: [mortality] explode all trees |
| 17 | Mortality: ti,ab,kw OR mortality NEXT rate:ti,ab,kw OR death:ti,ab,kw death NEXT rate:ti,ab,kw |
| 18 | Mesh Descriptor: [patient readmission] explode all trees |
| 19 | readmission:ti,ab,kw OR rehospitalization:ti,ab,kw |
| 20 | #14 OR #15 OR #16 OR #17 OR #18 OR #19 |
| 21 | #3 AND #10 AND #13 AND #20 = 105 trials |
|  | FILTERS: PUBYEAR 2000 TO 2023 AND ( LIMIT-TO ( DOCTYPE , "TRIALS" ) ) AND ( LIMIT-TO ( LANGUAGE , "English") |
|  | SOURCED FROM: PUBMED=71 EMBASE=63, CT.GOV=13, WHO ICTRP=1 |
|  | **EBSCOhost (**Academic Search Complete) 148, imported reference 140, 8 duplicates removed |
| 1 | AB (importance OR significance OR value or effect OR benefit OR impact OR advantage OR effectiveness OR efficacy) |
| 2 | AB (Nurses OR “registered nurse” OR “bedside nurse*” OR “stroke nurse*” OR “rehabilitation nurse*” OR “geriatric nurse*” OR “transition nurse*” OR nurse-led OR “transition coordinator” OR “nurse coach” OR “nurse coordinator” OR multidisciplinary OR “community nurse*” OR “head nurse” OR “charge nurse” OR “stroke survivor*” OR “stroke patient*” OR “stroke caregiver*” OR “patient* with stroke” OR “people with stroke”) |
| 3 | AB ("Transitional Care" OR "transition care" OR “patient transition*” OR “patient handoff” OR “patient transfer” OR “patient navigation” OR "continuity of patient care" OR "patient discharge" OR "patient transfer" OR "discharge home" OR "home discharge" OR "discharge planning" OR aftercare OR “post hospitalization” OR “stroke rehabilitation” OR “stroke recovery” OR “stroke intervention” or “post stroke” OR “stroke care”) |
| 4 | AB (“quality of life” OR mortality OR “mortality rate” OR death OR “death rate” OR “functional disability” OR disability OR “activities of daily living” OR “functional decline” OR “functional impairment” OR “physical function” OR “cognitive decline” OR “cognitive impairment” OR “health status” OR “sickness impact” OR readmission OR rehospitalisation) |
| 5 | AB (“randomized controlled trial” or RCT or “randomised control trial” or “randomized clinical trial” or “randomized controlled study”) |
| 6 | 1 AND 2 AND 3 AND 4 AND 5 = 143 |
|  | EBSCOhost (Open Dissertations)= 5 |
|  | Total 148 |
|  | **WEB OF SCIENCE CORE COLLECTION** |
| 1 | TS=(effect OR benefit OR impact OR effectiveness OR efficacy) |
| 2 | TS=(nurse OR "registered nurse" OR "stroke nurse" OR "rehabilitation nurse" OR "geriatric nurse" OR "transition nurse" OR nurse-led OR "nurse coach" OR "stroke survivor" OR "stroke patient") |
| 3 | TS=(stroke OR "cerebrovascular accident" OR "Cerebrovascular event") |
| 4 | TS=("continuity of care" OR "patient discharge" OR "discharge home" OR "post discharge" OR "hospital discharge" OR "discharge plan" OR "discharge planning" OR "Transitional care" OR "Patient transition" OR "patient handoff" OR "patient transfer" OR "patient navigation" OR "post stroke" OR "Stroke rehabilitation") |
| 5 | TS=("quality of life" OR mortality OR death OR disability OR "functional decline" OR "cognitive decline" OR “health status” OR “sickness impact” OR readmission) |
| 6 | 1 AND 2 AND 3 AND 4 AND 5 = 352 |
|  | FILTERS: (LIMIT-TO PUBYEAR “2000 TO 2023”) AND ( LIMIT-TO ( DOCTYPE , "article or clinical trial" ) ) AND ( LIMIT-TO ( LANGUAGE , "English" ) AND LIMIT-TO (open access) AND LIMIT-TO (Research areas; nursing or rehabilitation or geriatrics gerontology or neuroscience neurology or cardiovascular system cardiology or healthcare sciences services or Behaviral science or psychology) |
|  | <https://webofscience.clarivate.cn/wos/alldb/summary/2643cc6e-04c1-4f19-b4bd-a76f7ec40680-da2caf9c/relevance/1> |
|  | **SCIENCE DIRECT on Elsevier.com** |
| 1 | effect OR effectiveness |
| 2 | “post stroke” |
| 3 | Nurse |
| 3 | “continuity of care" OR "Transitional care" |
| 5 | "quality of life" OR mortality OR readmission |
| 6 | 1 AND 2 AND 3 AND 4= 58 |
|  | Limit Year: 2000-2023 |

**Supplementary File 1 Table 2: Full Text Review**

| SN | ARTICLES | DECISIONS |
| --- | --- | --- |
| 1. | Aidar, F.J., et al., A randomized trial investigating the influence of strength training on quality of life in ischemic stroke. Topics in Stroke Rehabilitation, 2016. 23(2): p. 84-89. 10.1080/10749357.2015.1110307 | exclude; not nurse-led transition care programs intervention |
| 2. | Allen, K., et al., A Randomized Trial Testing the Superiority of a Postdischarge Care Management Model for Stroke Survivors. JOURNAL OF STROKE & CEREBROVASCULAR DISEASES, 2009. 18(6): p. 443-452. 10.1016/j.jstrokecerebrovasdis.2009.02.002 | include |
| 3. | Andersen, H.E., et al., Can readmission after stroke be prevented? Results of a randomized clinical study: A postdischarge follow-up service for stroke survivors. Stroke, 2000. 31(5): p. 1038-1045. 10.1161/01.STR.31.5.1038 | exclude; not nurse-led transition care programs intervention |
| 4. | Aoki, Y. and K. Nakayama, Improving older adults stroke survivors’ decision-making when selecting a discharge location: A randomized controlled trial protocol. International Journal of Nursing Knowledge, 2023. 34(3): p. 185-192. 10.1111/2047-3095.12393 | exclude; no outcome of interest |
| 5. | Ashghali Farahani, M., et al., The effect of a supportive home care program on caregiver burden with stroke patients in Iran: an experimental study. BMC HEALTH SERVICES RESEARCH, 2021. 21(1). 10.1186/s12913-021-06340-4 | exclude; no population of interest |
| 6. | Auger, K.A., J.M. Simmons, and H.L. Tubbs-Cooley, Postdischarge Nurse Home Visits and Reuse: The Hospital to Home Outcomes (H2O) Trial. Pediatrics, 2018. 142(1): p. 1-10. 10.1542/peds.2017-3919 | exclude= ineligible population |
| 7. | Boter, H. and H.S. Grp, Multicenter Randomized controlled trial of an outreach nursing support program for recently discharged stroke patients. STROKE, 2004. 35(12): p. 2867-2872. 10.1161/01.STR.0000147717.57531.e5 | include |
| 8. | Bushnell, C.D., et al., A Person-Centered Approach to Poststroke Care: The COMprehensive Post-Acute Stroke Services Model. JOURNAL OF THE AMERICAN GERIATRICS SOCIETY, 2018. 66(5): p. 1025-1030. 10.1111/jgs.15322 | exclude; no outcome of interest |
| 9. | Chen, L., et al., Longitudinal Study of Effectiveness of a Patient-Centered Self-Management Empowerment Intervention During Predischarge Planning on Stroke Survivors. Worldviews on Evidence-Based Nursing, 2018. 15(3): p. 197-205. 10.1111/wvn.12295 | include |
| 10. | Chen L, S.J., Evaluation of a modified home care intervention for stroke patients in transition from hospital to home. Stroke, 2016. | exclude= quasi-experimental study design |
| 11. | Chen, Y., et al., A pragmatic randomized controlled trial of a cardiac hospital-to-home transitional care program in a Singapore academic medical center. Journal of Patient Safety & Risk Management, 2020. 25(2): p. 55-66. 10.1177/2516043520914196 | exclude= population of interest |
| 12. | Cho, S.J., et al., Effect of a Hospital-To-Home Transitional Intervention Based on an Interaction Model of Client Health Behavior for Adult Patients with Stroke. Journal of Community Health Nursing, 2023. 40(4): p. 273-288. 10.1080/07370016.2023.2227154 | exclude= no full text |
| 13. | Chu, K., et al., Feasibility of a Nurse-Trained, Family Member-Delivered Rehabilitation Model for Disabled Stroke Patients in Rural Chongqing, China. J Stroke Cerebrovasc Dis, 2020. 29(12): p. 105382. 10.1016/j.jstrokecerebrovasdis.2020.105382 | include |
| 14. | Condon, C., et al., Reducing Readmissions After Stroke With a Structured Nurse Practitioner/Registered Nurse Transitional Stroke Program. Stroke, 2016. 47(6): p. 1599-604. https://dx.doi.org/10.1161/STROKEAHA.115.012524 | exclude= pre and post-modification quality improvement study |
| 15. | Courtney, M.D., et al., Improved functional ability and independence in activities of daily living for older adults at high risk of hospital readmission: a randomized controlled trial. Journal of Evaluation in Clinical Practice, 2012. 18(1): p. 128-134. 10.1111/j.1365-2753.2010.01547.x | exclude; no outcome of interest |
| 16. | Day, C.B., et al., Nursing Home Care Intervention Post Stroke (SHARE) 1 year effect on the burden of family caregivers for older adults in Brazil: A randomized controlled trial. HEALTH & SOCIAL CARE IN THE COMMUNITY, 2021. 29(1): p. 56-65. 10.1111/hsc.13068 | exclude; no population of interest |
| 17. | Deen, T., et al., The Impact of Stroke Nurse Navigation on Patient Compliance Post discharge. Rehabilitation nursing: the official journal of the Association of Rehabilitation Nurses, 2018. 43(2): p. 65-72. https://dx.doi.org/10.1002/rnj.305 | exclude= design, chart review and longitudinal study |
| 18. | Deepradit, S., et al., Effectiveness of a family-based program for post-stroke patients and families: A cluster randomized controlled trial. International Journal of Nursing Sciences, 2023. 10(4): p. 446-455. 10.1016/j.ijnss.2023.09.020 | exclude; no outcome of interest |
| 19. | Demir Avci, Y. and S. Gözüm, Effects of Transitional Care Model-Based Interventions for Stroke Patients and Caregivers on Caregivers' Competence and Patient Outcomes: Randomized Controlled Trial. CIN - Computers Informatics Nursing, 2023. 41(10): p. 805-814. 10.1097/CIN.0000000000000991 | include |
| 20. | Devi, B., Impact of Nurse-Led Stroke Education Program (NSEP) on ADL and SS-QOL among Patients with Stroke and Burden among Caregivers. International Journal of Nutrition, Pharmacology, Neurological Diseases, 2022. 12(4): p. 253-262. 10.4103/ijnpnd.ijnpnd_47_22 | exclude = no access to full article |
| 21. | Donnelly, M., et al., Randomized controlled trial of an early discharge rehabilitation service: the Belfast Community Stroke Trial. Stroke, 2004. 35(1): p. 127-33. 10.1161/01.Str.0000106911.96026.8f | exclude= community-based multidisciplinary stroke team led |
| 22. | Duncan, P.W., et al., The Comprehensive Post-Acute Stroke Services (COMPASS) study: design and methods for a cluster-randomized pragmatic trial. BMC NEUROLOGY, 2017. 17. 10.1186/s12883-017-0907-1 | include |
| 23. | Eichner, F.A., et al., Trial design and pilot phase results of a cluster-randomised intervention trial to improve stroke care after hospital discharge – The structured ambulatory post-stroke care program (SANO). 2021. 6(2): p. 213-221. 10.1177/2396987320910596 | exclude= no outcome of interest |
| 24. | Ellis, G., et al., The impact of stroke nurse specialist input on risk factor modification: a randomised controlled trial. Age Ageing, 2005. 34(4): p. 389-92. 10.1093/ageing/afi075 | exclude= no outcome of interest |
| 25. | Ellis-Hill, C., et al., HeART of Stroke: randomised controlled, parallel-arm, feasibility study of a community-based arts and health intervention plus usual care compared with usual care to increase psychological well-being in people following a stroke. BMJ Open, 2019. 9(3): p. e021098. 10.1136/bmjopen-2017-021098 | exclude= setting, community |
| 26. | Fjærtoft, H., G. Rohweder, and B. Indredavik, Stroke unit care combined with early supported discharge improves 5-year outcome: A randomized controlled trial. Stroke, 2011. 42(6): p. 1707-1711. 10.1161/STROKEAHA.110.601153 | exclude= time for outcome of interest |
| 27. | Gamawa, A.U., et al., Effectiveness of an Integrated Education for Self-Management in Stroke Rehabilitation in Northeast Nigeria: A Randomized Controlled Trial. Pakistan Journal of Medical Research, 2021. 60(1): p. 12-19. | exclude= not transition care programs intervention |
| 28. | Geng, G., et al., Impact of transitional care for discharged elderly stroke patients in China: an application of the Integrated Behavioral Model. Topics in Stroke Rehabilitation, 2019. 26(8): p. 621-629. 10.1080/10749357.2019.1647650 | exclude= clinical controlled trial |
| 29. | Görlitz, R.A., A. Rashid, and C. Weinhardt, Stroke manager service for improved post-acute continuity of care. Health Policy and Technology, 2012. 1(3): p. 145-154. https://doi.org/10.1016/j.hlpt.2012.07.008 | exclude= not transition care programs intervention |
| 30. | Haynes, H.N., et al., Transitions of care for stroke and TIA. Journal of the American Association of Nurse Practitioners, 2015. 27(10): p. 558-67. https://dx.doi.org/10.1002/2327-6924.12219 | exclude= design; a retrospective analysis |
| 31. | He, Y., et al., Nurse-led rapid rehabilitation following mechanical thrombectomy in patients with acute ischemic stroke: A historical control study. Medicine, 2023. 102(28): p. e34232. https://dx.doi.org/10.1097/MD.0000000000034232 | exclude=not nurse-led transition care programs |
| 32. | Hustoft, M., et al., The effect of team collaboration and continuity of care on health and disability among rehabilitation patients: a longitudinal survey-based study from western Norway. QUALITY OF LIFE RESEARCH, 2019. 28(10): p. 2773-2785. 10.1007/s11136-019-02216-7 | exclude= design; a longitudinal survey-based |
| 33. | Irewall, A.-L., et al., Nurse-led, telephone-based secondary preventive follow-up benefits stroke/TIA patients with low education: a randomized controlled trial sub-study. TRIALS, 2019. 20. 10.1186/s13063-018-3131-4 | exclude= no outcome of interest |
| 34. | Jarbandhan, A., et al., Feasibility of a home-based physiotherapy intervention to promote post-stroke mobility: A randomized controlled pilot study. PLoS ONE, 2022. 17(3): p. 1-15. 10.1371/journal.pone.0256455 | exclude= no outcome of interest |
| 35. | Kamoen, O., et al., Stroke coach: a pilot study of a personal digital coaching program for patients after ischemic stroke. Acta neurologica Belgica, 2020. 120(1): p. 91-97. https://dx.doi.org/10.1007/s13760-019-01218-z | exclude= access to full article |
| 36. | Kao, A., et al., Do clinical nurse specialist led stroke follow-up clinics reduce post-stroke hospital readmissions and recurrent vascular events? Internal medicine journal, 2020. 50(10): p. 1202-1207. https://dx.doi.org/10.1111/imj.14707 | exclude= design, a chart review not RCT |
| 37. | Langstaff, C., et al., Enhancing community-based rehabilitation for stroke survivors: Creating a discharge link. Topics in Stroke Rehabilitation, 2014. 21(6): p. 510-519. 10.1310/tsr2106-510 | exclude= design, not RCT |
| 38. | Lin, S., et al., Nurse-led health coaching programme to improve hospital-to-home transitional care for stroke survivors: A randomised controlled trial. Patient education and counseling, 2022. 105(4): p. 917-925. https://dx.doi.org/10.1016/j.pec.2021.07.020 | include |
| 39. | Liu, C., et al., Association of formal and informal care with health-related quality of life and depressive symptoms: findings from the Caring for Adults Recovering from the Effects of Stroke study. DISABILITY AND REHABILITATION, 2021. 43(8): p. 1092-1100. 10.1080/09638288.2019.1650965 | exclude= design, not RCT |
| 40. | Liu, H. and V.W.Q. Lou, Functional recovery of older stroke patients discharged from hospital to home: The effects of cognitive status and different levels of therapy intensity. JOURNAL OF CLINICAL NURSING, 2019. 28(1-2): p. 47-55. 10.1111/jocn.14617 | exclude= design, not RCT A prospective follow-up evaluation |
| 41. | Lo, S.H.S., A.M. Chang, and J.P.C. Chau, Stroke self-management support improves survivors' self-efficacy and outcome expectation of self-management behaviors. Stroke, 2018. 49(3): p. 758-760. 10.1161/STROKEAHA.117.019437 | exclude= no outcome of interest |
| 42. | Lo, S.H.S., et al., Health Professional- and Volunteer-partnered Self-management Support (COMBO-KEY) to Promote Self-efficacy and Self-management Behaviors in People with Stroke: A Randomized Controlled Trial. Annals of Behavioral Medicine, 2023. 57(10): p. 866-876. 10.1093/abm/kaad028 | exclude= context, community based study |
| 43. | Lopez-Espuela, F., et al., Determinants of Quality of Life in Stroke Survivors After 6 Months, from a Comprehensive Stroke Unit: A Longitudinal Study. Biological Research for Nursing, 2015. 17(5): p. 461-468. 10.1177/1099800414553658 | exclude= design |
| 44. | Low, L.L., et al., Applying the Integrated Practice Unit Concept to a Modified Virtual Ward Model of Care for Patients at Highest Risk of Readmission: A Randomized Controlled Trial. PLoS ONE, 2017. 12(1): p. 1-18. 10.1371/journal.pone.0168757 | exclude= participants of interest |
| 45. | M, M.D., Using patient activation to transition patients from hospital to home. Stroke, 2015. | exclude= design, not RCT |
| 46. | Markle-Reid, M., et al., An integrated hospital-to-home transitional care intervention for older adults with stroke and multimorbidity: A feasibility study. Journal of comorbidity, 2020. 10: p. 2235042X19900451. https://dx.doi.org/10.1177/2235042X19900451 | exclude= no outcome of interest |
| 47. | McClain, J.V.t. and E.A. Chance, The Advanced Practice Nurse Will See You Now: Impact of a Transitional Care Clinic on Hospital Readmissions in Stroke Survivors. Journal of nursing care quality, 2020. 35(2): p. 147-152. https://dx.doi.org/10.1097/NCQ.0000000000000414 | exclude =chart review |
| 48. | McLachlan, A., A. Kerr, and M. Lee, A nurse-led and medically supported outpatient follow-up model following an acute coronary syndrome is as safe and effective as medical follow-up alone (ANZACS-QI 69. The New Zealand medical journal, 2022. 135(1563): p. 12-28. | exclude =participants of interest |
| 49. | Mitchell, E., et al., Implementation of an Advanced Practice Registered Nurse-Led Clinic to Improve Follow-up Care for Post-Ischemic Stroke Patients. The Journal of neuroscience nursing: journal of the American Association of Neuroscience Nurses, 2022. 54(5): p. 193-198. https://dx.doi.org/10.1097/JNN.0000000000000670 | exclude =design; A pre/post intervention design |
| 50. | Mohammadi, E., F. Hassandoost, and H. Mozhdehipanah, Evaluation of the “partnership care model” on quality of life and activity of daily living in stroke patients: A randomized clinical trial. Japan Journal of Nursing Science, 2022. 19(1). 10.1111/jjns.12448 | include |
| 51. | Montgomery, P., et al., Community reintegration of stroke survivors: the effect of a community navigation intervention. Journal of advanced nursing, 2015. 71(1): p. 214-225. 10.1111/jan.12471 | exclude = mixed-method with cohort and focused ethnography |
| 52. | Mou, H., S.K.K. Lam, and W.T. Chien, The effects of a family-focused dyadic psychoeducational intervention for stroke survivors and their family caregivers: A randomised controlled trial. International Journal of Nursing Studies, 2023. 143. 10.1016/j.ijnurstu.2023.104504 | include |
| 53. | Nelson, M.M., et al., Declining Patient Functioning and Caregiver Burden/Health: The Minnesota Stroke Survey-Quality of Life After Stroke Study. GERONTOLOGIST, 2008. 48(5): p. 573-583. 10.1093/geront/48.5.573 | exclude=not RCT |
| 54. | Nir, Z., Z. Zolotogorsky, and H. Sugarman, Structured nursing intervention versus routine rehabilitation after stroke. Am J Phys Med Rehabil, 2004. 83(7): p. 522-9. 10.1097/01.phm.0000130026.12790.20 | exclude= no outcome of interest |
| 55. | Nunes, H.J.M. and P.J.P. Queiros, Patient with stroke: hospital discharge planning, functionality and quality of life. Revista brasileira de enfermagem, 2017. 70(2): p. 415-423. 10.1590/0034-7167-2016-0166 | exclude= design; not RCT integrative literature review |
| 56. | Olaiya, M.T., et al., Effectiveness of a shared team approach between nurses and doctors for improved risk factor management in survivors of stroke: a cluster randomized controlled trial. European journal of neurology, 2017. 24(7): p. 920-928. https://dx.doi.org/10.1111/ene.13306 | exclude= no outcome of interest |
| 57. | Olson, D.M. and S.B. Juengst, The Hospital to Home Transition Following Acute Stroke. Nursing Clinics of North America, 2019. 54(3): p. 385-397. https://doi.org/10.1016/j.cnur.2019.04.007 | exclude= design; not RCT |
| 58. | Oquendo, B., et al., Better functional recovery after acute stroke in older patients managed in a new dedicated post-stroke geriatric unit compared to usual management. Journal of Nutrition, Health and Aging, 2024. 28(4). 10.1016/j.jnha.2023.100033 | exclude= design; not RCT a cohort observational |
| 59. | Quinn, E.B., et al., Effect of a telephone and web-based problem-solving intervention for stroke caregivers on stroke patient activities of daily living: A randomized controlled trial. Clinical rehabilitation, 2023. 37(8): p. 1062-1073. https://dx.doi.org/10.1177/02692155231157301 | exclude= no outcome of interest, not transition care programs |
| 60. | Rafsten, L., et al., Gothenburg Very Early Supported Discharge study (GOTVED): a randomised controlled trial investigating anxiety and overall disability in the first year after stroke. BMC Neurol, 2019. 19(1): p. 277. 10.1186/s12883-019-1503-3 | exclude= no outcome of interest, MDT led |
| 61. | Reeves, M.J., B. Boden-Albala, and D.A. Cadilhac, Care Transition Interventions to Improve Stroke Outcomes: Evidence Gaps in Underserved and Minority Populations. STROKE, 2023. 54(2): p. 386-395. 10.1161/STROKEAHA.122.039565 | exclude= design; not RCT |
| 62. | Reid, M., et al., Implementation and evaluation of an integrated hospital-to-home transitional care intervention for older adults with stroke and multimorbidity: a feasibility study. International Journal of Integrated Care (IJIC), 2019. 19(S1): p. 1-2. 10.5334/ijic.s3367 | exclude= no outcome of interest |
| 63. | Roderick, P., et al., Stroke rehabilitation after hospital discharge: a randomized trial comparing domiciliary and day-hospital care. AGE AND AGEING, 2001. 30(4): p. 303-310. 10.1093/ageing/30.4.303 | exclude=not nurse-led transition care programs |
| 64. | Rodgers, H., et al., Evaluation of an extended stroke rehabilitation service (EXTRAS) a randomized controlled trial and economic analysis. Stroke, 2019. 50(12): p. 3561-3568. 10.1161/STROKEAHA.119.024876 | exclude= no outcome of interest |
| 65. | Rosenberg, K., NP-Led Clinic Reduces 30-Day Stroke Readmissions. The American journal of nursing, 2016. 116(8): p. 53. https://dx.doi.org/10.1097/01.NAJ.0000490177.30017.ad | exclude=design not RCT |
| 66. | Rymer, J.A., et al., Advanced Practice Provider Versus Physician-Only Outpatient Follow-Up After Acute Myocardial Infarction. Journal of the American Heart Association, 2018. 7(17): p. e008481. https://dx.doi.org/10.1161/JAHA.117.008481 | exclude=population of interest |
| 67. | Saal, S., et al., Effect of a stroke support service in Germany: A randomized trial. Topics in Stroke Rehabilitation, 2015. 22(6): p. 429-436. 10.1179/1074935714Z.0000000047 | exclude=no access to full article |
| 68. | Schwarzbach, C.J., et al., The structured ambulatory post-stroke care program for outpatient aftercare in patients with ischaemic stroke in Germany (SANO): an open-label, cluster-randomised controlled trial. Lancet Neurol, 2023. 22(9): p. 787-799. 10.1016/s1474-4422(23)00216-8 | exclude=no outcome of interest |
| 69. | Shaughnessy, M. and F.W. Whitney, Post-stroke consultation service: A nurse-managed model for care delivery. Topics in Stroke Rehabilitation, 2007. 14(2): p. 43-48. 10.1310/tsr1402-43 | exclude=no outcome of interest, not RCT |
| 70. | Shyu, Y.L., et al., A clinical trial of an individualised intervention programme for family caregivers of older stroke victims in Taiwan. Journal of Clinical Nursing, 2010. 19(11-12): p. 1675-1685. 10.1111/j.1365-2702.2009.03124.x | exclude=no participants of interest |
| 71. | Smith, J., A. Forster, and J. Young, A randomized trial to evaluate an education programme for patients and carers after stroke. Clinical Rehabilitation, 2004. 18(7): p. 726-736. 10.1191/0269215504cr790oa | exclude=not nurse led transition care programs intervention, no outcome of interest |
| 72. | Sulch, D., et al., Randomized controlled trial of Integrated (managed) Care Pathway for stroke rehabilitation. Stroke, 2000. 31(8): p. 1929-1934. 10.1161/01.STR.31.8.1929 | exclude=not nurse led transition care programs intervention |
| 73. | Swank, C., et al., The impact of a patient-directed activity program on functional outcomes and activity participation after stroke during inpatient rehabilitation—a randomized controlled trial. Clinical Rehabilitation, 2020. 34(4): p. 504-514. 10.1177/0269215519901153 | exclude=not nurse led transition care programs intervention |
| 74. | Ugur, H.G. and B. Erci, THE EFFECT OF HOME CARE FOR STROKE PATIENTS AND EDUCATION OF CAREGIVERS ON THE CAREGIVER BURDEN AND QUALITY OF LIFE. ACTA CLINICA CROATICA, 2019. 58(2): p. 321-332. 10.20471/acc.2019.58.02.16 | exclude=context home care |
| 75. | Van Den Berg, M., et al., Early supported discharge by caregiver-mediated exercises and e-health support after stroke: A proof-of-concept trial. Stroke, 2016. 47(7): p. 1885-1892. 10.1161/STROKEAHA.116.013431 | exclude=not nurse led transition care programs intervention |
| 76. | Van Der Vlegel-Brouwer, W., et al., Evaluating a transitional care programme for the average and the oldest old: a presentation of results from the quantitative phase of a mixed methods study. International Journal of Integrated Care (IJIC), 2022. 22: p. 1-2. 10.5334/ijic.ICIC21056 | exclude= design; mixed methods study |
| 77. | Van Spall, H. and M.K. Ong, 2016 - Transition care with telemonitoring did not reduce readmission after hospitalization for heart failure. ACP Journal Club, 2016. 164(10): p. 10-10. 10.7326/ACPJC-2016-164-10-059 | exclude=no full access |
| 78. | Vanacker, P., et al., An individualized coaching program for patients with acute ischemic stroke: Feasibility study. Clinical neurology and neurosurgery, 2017. 154: p. 89-93. https://dx.doi.org/10.1016/j.clineuro.2017.01.017 | exclude= no outcome of interest, not RCT, feasibility study |
| 79. | Verberne, D.P.J., et al., Nurse-led stroke aftercare addressing long-term psychosocial outcome: a comparison to care-as-usual. Disability and rehabilitation, 2022. 44(12): p. 2849-2857. https://dx.doi.org/10.1080/09638288.2020.1849417 | exclude comparative effectiveness research design |
| 80. | Vluggen, T., et al., Effectiveness of an integrated multidisciplinary geriatric rehabilitation programme for older persons with stroke: a multicentre randomised controlled trial. BMC Geriatr, 2021. 21(1): p. 134. 10.1186/s12877-021-02082-4 | exclude=context, nursing home |
| 81. | von Koch, L., et al., A randomized controlled trial of rehabilitation at home after stroke in Southwest Stockholm: outcome at six months. Scand J Rehabil Med, 2000. 32(2): p. 80-6. 10.1080/003655000750045596 | exclude=no access to full article |
| 82. | Wang, J., et al., Impact of rehabilitation adherence and depressive symptoms on post-stroke self-care ability and quality of life: a longitudinal study. Topics in Stroke Rehabilitation, 2023. 10.1080/10749357.2023.2259652 | exclude= design a longitudinal study |
| 83. | Wong, F.K.Y., et al., Effects of a transitional home-based care program for stroke survivors in Harbin, China: a randomized controlled trial. Age & Ageing, 2022. 51(2): p. 1-10. 10.1093/ageing/afac027 | include |
| 84. | Wong, F.K.Y. and S.M. Yeung, Effects of a 4-week transitional care programme for discharged stroke survivors in Hong Kong: a randomised controlled trial. Health & social care in the community, 2015. 23(6): p. 619-31. https://dx.doi.org/10.1111/hsc.12177 | include |
| 85. | Xu, X.Y., et al., Impact of extended nursing model after multi-disciplinary treatment on young patient with post-stroke. World J Clin Cases, 2023. 11(14): p. 3148-3157. 10.12998/wjcc.v11.i14.3148 | exclude is case control study |
| 86. | Yan, L.L., et al., A randomized controlled trial on rehabilitation through caregiver-delivered nurse-organized service programs for disabled stroke patients in rural china (the RECOVER trial): design and rationale. International Journal of Stroke, 2016. 11(7): p. 823-830. 10.1177/1747493016654290 | exclude=no ouctome of interest |
| 87. | Yue, S., X. Jiang, and T. Wong, Effects of a nurse-led acupressure programme for stroke patients in China. Journal of clinical nursing, 2013. 22(7-8): p. 1182-8. https://dx.doi.org/10.1111/j.1365-2702.2012.04127.x | exclude= not nurse-led transition care programs |
| 88. | Zhang, L., et al., Effects of Coaching-Based Teleoccupational Guidance for Home-Based Stroke Survivors and Their Family Caregivers: A Pilot Randomised Controlled Trial. INTERNATIONAL JOURNAL OF ENVIRONMENTAL RESEARCH AND PUBLIC HEALTH, 2022. 19(23). 10.3390/ijerph192316355 | exclude=context, home based |
| 89. | Zhou, B., et al., Caregiver-Delivered Stroke Rehabilitation in Rural China: The RECOVER Randomized Controlled Trial. Stroke, 2019. 50(7): p. 1825-1830. 10.1161/STROKEAHA.118.021558 | exclude= design cluster, intervention is not direct nurse patient |
| **HAND SEARCH** | | |
| 1. | Allen, K.R., et al., Effectiveness of a postdischarge care management model for stroke and transient ischemic attack: a randomized trial. J Stroke Cerebrovasc Dis, 2002. 11(2): p. 88-98. 10.1053/jscd.2002.127106 | include |
| 2. | Askim, T., et al., Evaluation of an extended stroke unit service with early supported discharge for patients living in a rural community. A randomized controlled trial. Clin Rehabil, 2004. 18(3): p. 238-48. 10.1191/0269215504cr752oa | include |
| 3. | Burton, C. and B. Gibbon, Expanding the role of the stroke nurse: a pragmatic clinical trial. J Adv Nurs, 2005. 52(6): p. 640-50. 10.1111/j.1365-2648.2005.03639.x | include |
| 4. | Chalermwannapong, S., et al. Effects of the transitional care program on functional ability and quality of life of stroke survivors. 2010. | include |
| 5. | Fjaertoft, H., et al., Acute stroke unit care combined with early supported discharge. Long-term effects on quality of life. A randomized controlled trial. Clin Rehabil, 2004. 18(5): p. 580-6. 10.1191/0269215504cr773oa | include |
| 6. | Liu, L., Effects of Transitional Care on Functional Exercise: Compliance and Health Status of Stroke Patients. Acta Medica Mediterranea, 2028. 34(959). | include |
| 7. | Mayo, N.E., et al., Bridging the gap: the effectiveness of teaming a stroke coordinator with patient's personal physician on the outcome of stroke. Age Ageing, 2008. 37(1): p. 32-8. 10.1093/ageing/afm133 | include |


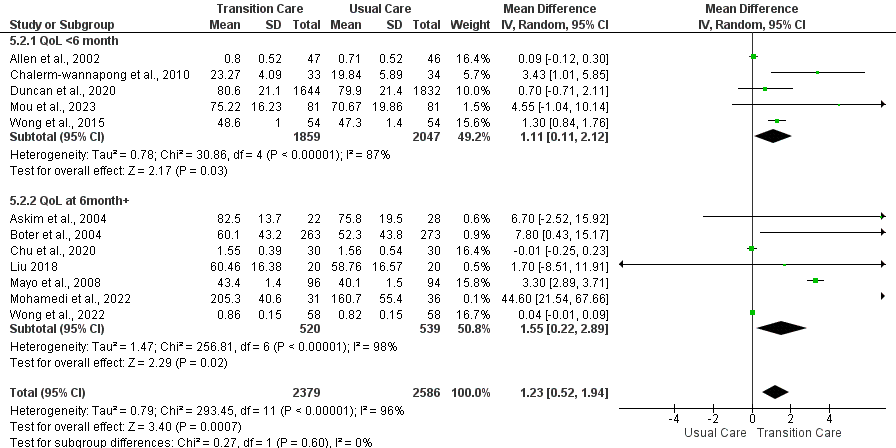


Supplementary File 1 Figure 1: The subgroup analysis on the effect of nurse-led transition care programs on stroke survivors’ quality of life


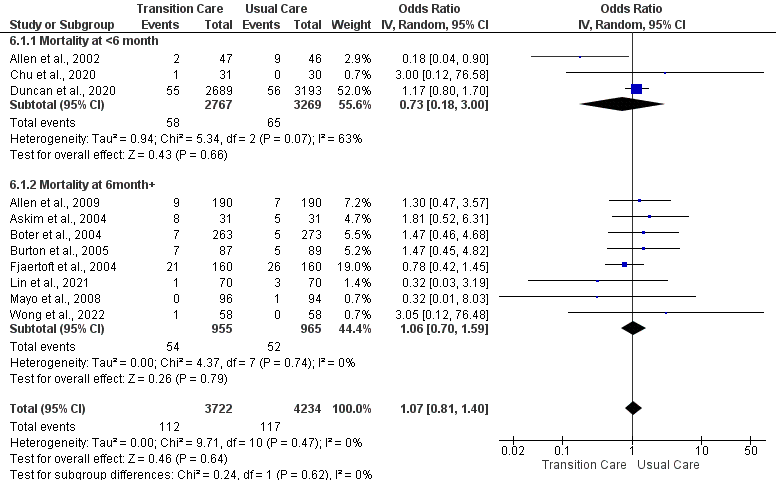


Supplementary File 1 Figure 2: The subgroup analysis of the effect of nurse-led transition care programs on mortality


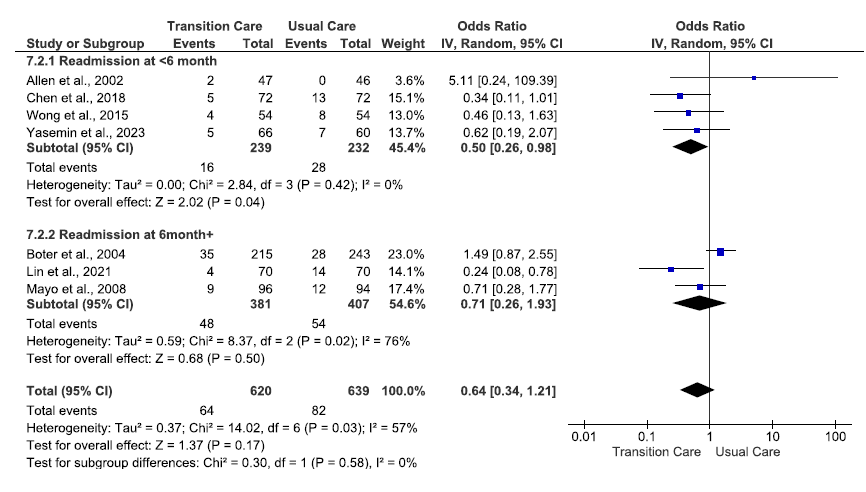


Supplementary File 1 Figure 3: The subgroup analysis of the effect of nurse-led transition care programs on readmission


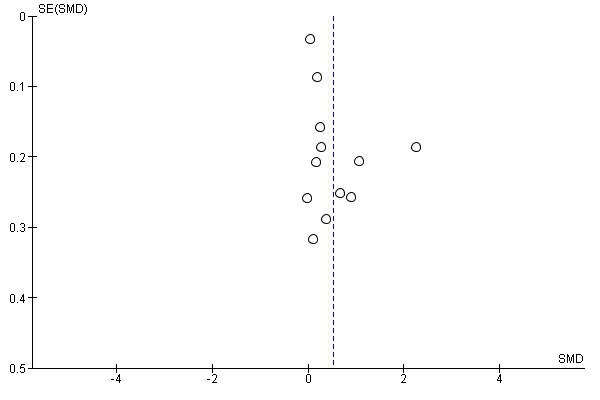


Supplementary File 1 Figure 4a: Funnel plot for quality of life


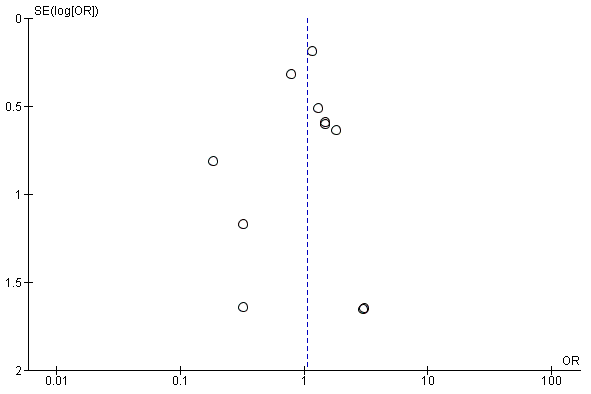


Supplementary File 1 Figure 4b: Funnel plot for mortality


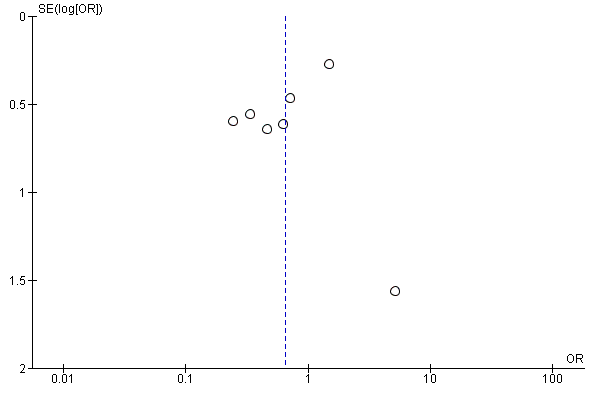


Supplementary File 1 Figure 4c: Funnel plot for readmission


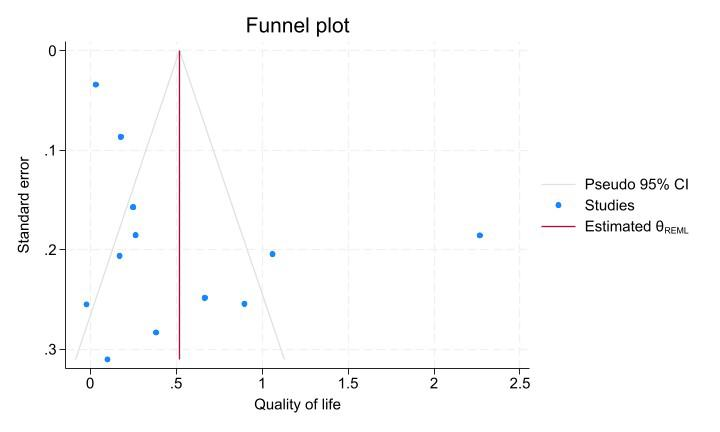


Supplementary File 1 Figure 5a: Funnel plot on trim-and-fill method for quality of life


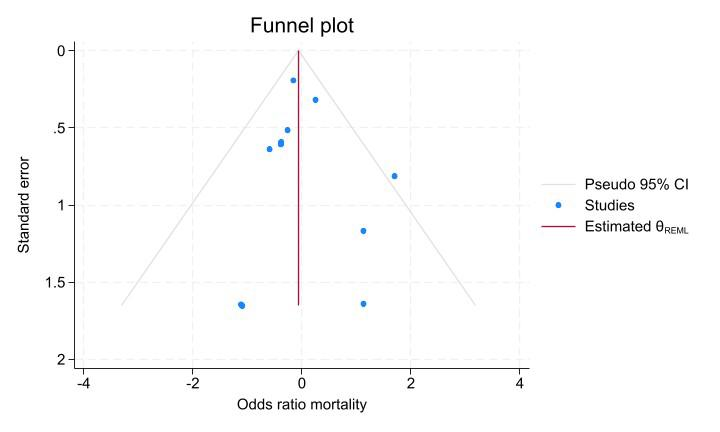


Supplementary File 1 Figure 5b: Funnel plot on trim-and-fill method for mortality


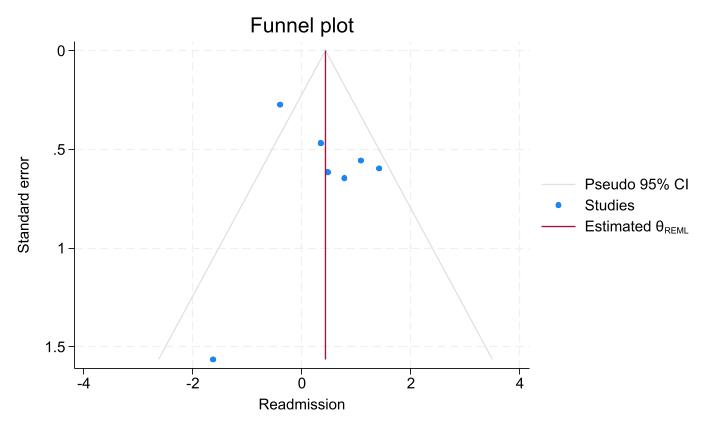


Supplementary File 1 Figure 5c: Funnel plot on trim-and-fill method for readmission


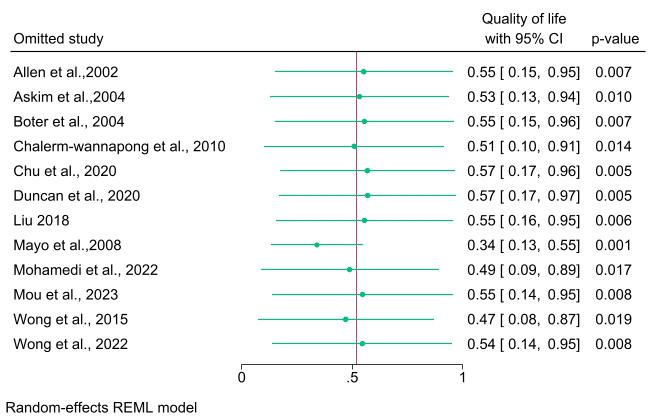


Supplementary File 1 Figure 6a: Sensitivity analysis using leave-one-out method for quality of life


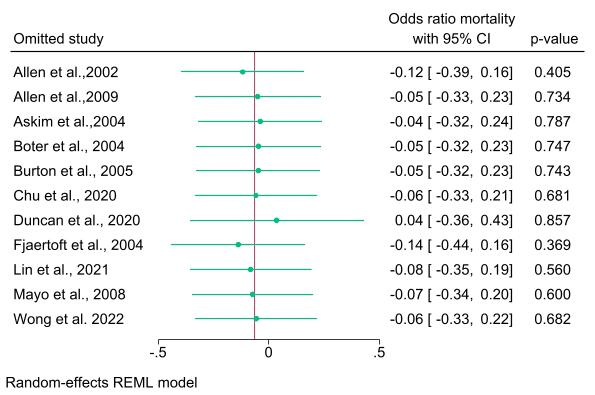


Supplementary File 1 Figure 6b: Sensitivity analysis using leave-one-out method for mortality


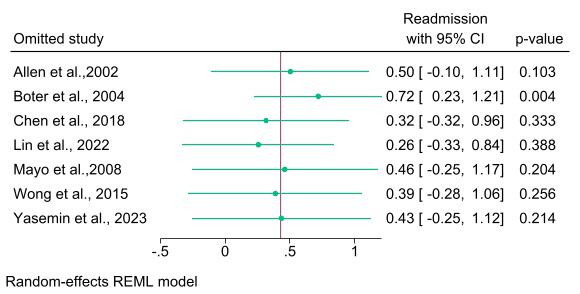


Supplementary File 1 Figure 6c: Sensitivity analysis using leave-one-out method for readmission
